# Driving pressure of respiratory system and lung stress in mechanically ventilated patients with active breathing

**DOI:** 10.1186/s13054-024-04797-3

**Published:** 2024-01-12

**Authors:** Vaia Stamatopoulou, Evangelia Akoumianaki, Katerina Vaporidi, Efstathios Stamatopoulos, Eumorfia Kondili, Dimitrios Georgopoulos

**Affiliations:** 1 Intensive Care Medicine Department, University Hospital of Heraklion, Heraklion, Crete Greece; 2 Medical School, University of Crete, Heraklion, Crete Greece; 3 Decision Support Systems, Laboratory, School of Electrical and Computer Engineering, National Technical University of Athens, Athens, Greece

## Abstract

**Background:**

During control mechanical ventilation (CMV), the driving pressure of the respiratory system (Δ*P*_rs_) serves as a surrogate of transpulmonary driving pressure (Δ*P*_lung_). Expiratory muscle activity that decreases end-expiratory lung volume may impair the validity of Δ*P*_rs_ to reflect Δ*P*_lung_. This prospective observational study in patients with acute respiratory distress syndrome (ARDS) ventilated with proportional assist ventilation (PAV+), aimed to investigate: (1) the prevalence of elevated Δ*P*_lung_, (2) the Δ*P*_rs_-Δ*P*_lung_ relationship, and (3) whether dynamic transpulmonary pressure (Plung_sw_) and effort indices (transdiaphragmatic and respiratory muscle pressure swings) remain within safe limits.

**Methods:**

Thirty-one patients instrumented with esophageal and gastric catheters (*n* = 22) were switched from CMV to PAV+ and respiratory variables were recorded, over a maximum of 24 h. To decrease the contribution of random breaths with irregular characteristics, a 7-breath moving average technique was applied. In each patient, measurements were also analyzed per deciles of increasing lung elastance (*E*_lung_). Patients were divided into Group A, if end-inspiratory transpulmonary pressure (*P*_LEI_) increased as *E*_lung_ increased, and Group B, which showed a decrease or no change in *P*_LEI_ with *E*_lung_ increase.

**Results:**

In 44,836 occluded breaths, Δ*P*_lung_ ≥ 12 cmH_2_O was infrequently observed [0.0% (0.0–16.9%) of measurements]. End-expiratory lung volume decrease, due to active expiration, was associated with underestimation of Δ*P*_lung_ by Δ*P*_rs_, as suggested by a negative linear relationship between transpulmonary pressure at end-expiration (*P*_LEE_) and Δ*P*_lung_/Δ*P*_rs_. Group A included 17 and Group B 14 patients. As *E*_lung_ increased, Δ*P*_lung_ increased mainly due to *P*_LEI_ increase in Group A, and *P*_LEE_ decrease in Group B. Although Δ*P*_rs_ had an area receiver operating characteristic curve (AUC) of 0.87 (95% confidence intervals 0.82–0.92, *P* < 0.001) for Δ*P*_lung_ ≥ 12 cmH_2_O, this was due exclusively to Group A [0.91 (0.86–0.95), *P* < 0.001]. In Group B, Δ*P*_rs_ showed no predictive capacity for detecting Δ*P*_lung_ ≥ 12 cmH_2_O [0.65 (0.52–0.78), *P* > 0.05]. Most of the time Plung_sw_ and effort indices remained within safe range.

**Conclusion:**

In patients with ARDS ventilated with PAV+, injurious tidal lung stress and effort were infrequent. In the presence of expiratory muscle activity, Δ*P*_rs_ underestimated Δ*P*_lung_. This phenomenon limits the usefulness of Δ*P*_rs_ as a surrogate of tidal lung stress, regardless of the mode of support.

**Supplementary Information:**

The online version contains supplementary material available at 10.1186/s13054-024-04797-3.

## Introduction

Transpulmonary driving pressure (Δ*P*_lung_) represents a direct measurement of static tidal lung stress and is proportional to lung strain, key mediators of ventilator-induced lung injury [1–4]. Despite its importance during mechanical ventilation, its clinical use remains limited due to the need for esophageal catheter insertion [5]. For this reason, during passive mechanical ventilation, the driving pressure of the respiratory system (Δ*P*_rs_), which is calculated as the difference between end-inspiratory plateau pressure (*P*_plat_) and total positive end-expiratory pressure (PEEP), is used as a surrogate for Δ*P*_lung_. Indeed, Δ*P*_rs_ can reliably predict increased Δ*P*_lung_, with high Δ*P*_rs_ (≥ 15 cmH_2_O) being associated with elevated morbidity and mortality [2, 6–8].

**Fig. 1:**
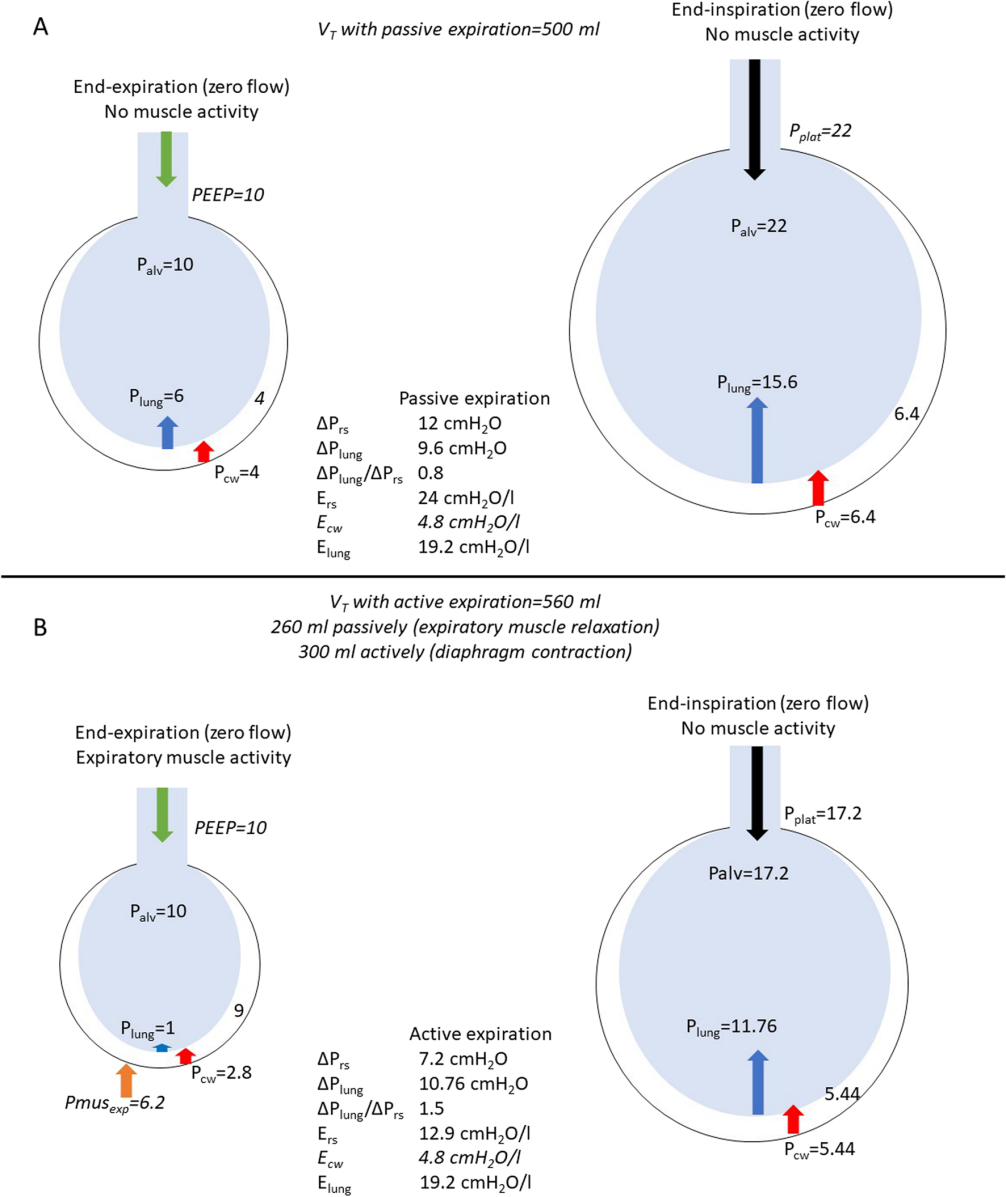
Effect of decreasing end-expiratory lung volume below (*V*_EE<FRC_) that corresponding to PEEP (*V*_EE,PEEP_) on calculation of driving pressure of respiratory system (Δ*P*_rs_) and lung (Δ*P*_lung_). Lung (*E*_lung_) and chest wall (*E*_cw_) elastance were kept constant at all lung volumes. Blue and white circles denote lung and chest wall, respectively. Set values are shown using italics. The numbers between the circles represent pleural pressure (*P*_pl_). Arrows show the magnitude of PEEP, end-inspiratory plateau pressure (*P*_plat_), elastic recoil pressure of chest wall (*P*_cw_) and lung (*P*_lung_), and expiratory muscle pressure (Pmus_exp_). Panel **A** shows applied pressures (cmH_2_O) when expiration is passive. Tidal volume (*V*_T_) is set to 500ml, *P*_pl_ at end-expiration to 4 cmH_2_O and *P*_plat_ to 22 cmH_2_O. Δ*P*_rs_ = *P*_plat_-PEEP = 12 cmH_2_O and respiratory system elastance (*E*_rs_) = Δ*P*_rs_/*V*_T_ = 12/0.5 = 24 cmH_2_O/l. *E*_cw_ is set to 20% of *E*_rs_ (4.8 cmH_2_O/l). At end-expiration, alveolar pressure (*P*_alv_) = PEEP, *P*_cw_ = *P*_pl_ = 4 cmH_2_O and *P*_lung_ = *P*_alv_-*P*_pl_ = 6 cmH_2_O. Notice that *P*_alv_ = *P*_lung_ + *P*_cw_. At end-inspiration *P*_pl_ increases to 6.4 cmH_2_O (4 + *E*_cw_ × *V*_T_ = 4 + 2.4 = 6.4), *P*_cw_ = 6.4 cmH_2_O and *P*_l__ung_ = *P*_alv_-*P*_pl_ = 22–6.4 = 15.6 cmH_2_O. Δ*P*_lung_ = 15.6–6 = 9.6 cmH_2_O and *E*_lung_ = Δ*P*_lung_/*V*_T_ = 9.6/0.5 = 19.2 cmH_2_O/l. Panel **B** shows pressures when expiration is active. Because of expiratory muscle activity, *V*_EE<FRC_ is set to 260 ml and therefore, compared to passive expiration, *P*_cw_ decreases by 1.2 cmH_2_O (*E*_cw_ × 0.26). At end-expiration, Pmus_exp_ is set to 6.2 cmH_2_O and *P*_pl_ is 9 cmH_2_O (*P*_pl_ = *P*_cw_ + Pmus_exp_). *P*_lung_ = *P*_alv_-*P*_pl_ = 10–9 = 1 cmH_2_O. Transdiaphragmatic pressure (Pdi) is deemed similar to A and begins to rise when flow is expiratory, before the full relaxation of expiratory muscles. Assuming that Pdi increases volume above *V*_EE,PEEP_ by 300ml (only a portion of Pdi increases volume above *V*_EE,PEEP_), *V*_T_ is 560 ml. At end-inspiration, *P*_plat_ = PEEP plus the increase in elastic recoil pressure of respiratory system due to 300 ml increase in volume above *V*_EE,PEEP_ (*P*_plat_ = PEEP + 0.3 × 24 = 17.2 cmH_2_O). Δ*P*_rs_ = *P*_plat_-PEEP = 7.2 cmH_2_O and calculated *E*_rs_ = 7.2/0.56 = 12.9 cmH_2_O/l, underestimated by 46%, because Δ*P*_rs_ should be divided by 0.3 (the volume inflated above PEEP). *P*_cw_ is 5.44 cmH_2_O, 1.44 cmH_2_O higher than that at *V*_EE,PEEP_ (0.3 × 4.8 = 1.44). *P*_lung_ = *P*_alv_-*P*_pl_ = 17.2–5.44 = 11.76 cmH_2_O, Δ*P*_lung_ = 11.76–1 = 10.76 cmH_2_O and *E*_lung_ = Δ*P*_lung_/*V*_T_ = 19.2 cmH_2_O/l, similar to that in A. See Fig. S1 in the Additional file 1 for detailed further explanation

In mechanically ventilated patients with active breathing, measurement of Δ*P*_rs_ is challenging for two reasons. Firstly, *P*_plat_ calculation requires end-inspiratory occlusions during which respiratory muscle activity should be absent, which is often not the case during conventional assisted ventilation [9]. Proportional assist ventilation with load adjustable gain factors (PAV+), automatically performs end-inspiratory occlusions to measure *P*_plat_. The interference of respiratory muscle activity with *P*_plat_ calculation is largely minimized with this mode, because the end of mechanical inflation follows the end of neural inspiration [10]. Secondly, expiratory muscle activity is often observed in critically ill patients, potentially lowering end-expiratory lung volume below the level corresponding to PEEP [11–13]. As a result, the relaxation of expiratory muscles contributes to tidal volume (*V*_T_) [14–16]. This is a reflex protective mechanism, which at increased demands, increases *V*_T_ at the same end-inspiratory lung stress [14, 16]. Under these conditions Δ*P*_rs_, which assumes that the starting point of inflation is PEEP, does not account for the decrease in end-expiratory lung volume below the level corresponding to PEEP, leading to an underestimation of Δ*P*_lung_ (Fig. 1 and Additional file 1: Fig. S1). Unfortunately, this later issue is largely ignored in the literature.

Studies have shown that, in critically ill patients ventilated with PAV+, which via the control of breathing mechanisms permits the patients to determine *V*_T_ [10], Δ*P*_rs_ can be effectively maintained low [15, 17]. However, these studies did not measure Δ*P*_lung_. It is unknown whether Δ*P*_rs_ reliably predicts tidal static lung stress in patients with active expiration that lowers end-expiratory lung volume below that corresponding to PEEP. The primary aim of this study was to determine the occurrence of injurious tidal lung stress, as expressed by a high Δ*P*_lung_ (≥ 12 cmH_2_O) [18] in patients with acute respiratory distress syndrome (ARDS) ventilated with PAV+, and to elucidate the relationship between Δ*P*_rs_ and Δ*P*_lung_. We hypothesize that in a given patient, the decrease in end-expiratory lung volume, secondary to expiratory muscle contraction because of increased demands, can lead to underestimation of Δ*P*_lung_ by Δ*P*_rs_ to an unknown extent. A secondary objective was to explore if dynamic transpulmonary pressure swings (Plung_sw_) and indices of respiratory effort, reflected by transdiaphragmatic (ΔPdi) and respiratory muscles pressure (Pmus_sw_) swings, remain within a safe range. It was deemed safe to have values for Plung_sw_, ΔPdi, and Pmus_sw_ of less than 20, 3–12, and 3–15 cmH_2_O, respectively [19–22].

## Methods

This prospective observational study was conducted in the medical–surgical intensive care unit (ICU) of the University Hospital of Heraklion. The study was approved by the Hospital Ethics Committee (339/09/20-03-2019), and since there was no interference with patients’ management, signed informed consent was waived.

### Patients

Eligible for inclusion were intubated patients, admitted to the ICU for management of ARDS, and instrumented with esophageal and gastric catheters (NutriVent™) or only an esophageal catheter (Cooper-Surgical esophageal balloon kit) for clinical purposes. The patients were included at any time the treating physician switched them from control modes to PAV+ (Puritan-Bennett 840 ventilator, Medtronic, Boulder, CO) and estimated that they would remain on assisted mechanical ventilation for at least 24 h. The recording period was approximately 24 h, unless the patient was switched to other modes, placed on a T-piece earlier, or the recording was interrupted for procedural reasons. Patients who remained on PAV+ for less than 1 h were excluded from the analysis.

### Measurements: analysis

Airflow (V′), volume and airway (Paw), esophageal (Pes), gastric (Pgas), dynamic transpulmonary (*P*_lung_ = Paw-Pes), and transdiaphragmatic (Pdi = Pgas-Pes) pressures were monitored continuously. Using a customized computer program, all breaths with 300-ms end-inspiratory occlusions were identified and the beginning (zero flow) and end of inspiration (end of 300-ms occlusion) were marked. At these two points, Paw and Pes were measured and various respiratory variables, including static transpulmonary pressures and Pmus_sw_, were calculated using standard formulas [18, 23, 24]. Plung_sw_ and Pmus_sw_ during the breath were measured as the difference between the peak and nadir values. Expiratory muscle activity was estimated in the preceding breath by measuring the rise in Pgas (ΔPgas) during the expiratory phase [25, 26]. Each recording underwent a thorough examination to identify artifacts mainly due to esophageal peristalsis and issues related to improper balloon filling and position.

Significant expiratory muscle activity during expiration was determined by either an average ΔPgas > 1 cmH_2_O over the recording time or, in patients without gastric catheters, by a thorough examination of expiratory flow and Pes waveforms, which unequivocally demonstrated signs of active expiration [27]. Dynamic intrinsic PEEP (PEEPi) was calculated only in patients in whom both Pes and Pgas were available, as described previously [28].

In order to decrease the contribution of random breaths with irregular characteristics on the measured values, a seven-breath moving average (7-brMA) technique was performed and the results of this analysis are reported. Furthermore, in each patient all the artifact-free 7-brMA measurements were divided into deciles based on progressive increase in lung elastance (*E*_lung_) (Decile 1: the lowest range of *E*_lung_, Decile 10; the highest range of *E*_lung_, see Additional file 2 for reasoning of choosing *E*_lung_ to characterize deciles of 7-brMA measurements). Patients were divided into two groups (A and B), depending on how their end-inspiratory transpulmonary pressure (*P*_LEI_) responded to an increase in *E*_lung_, with the assumption that expiratory muscle contraction could, as a reflex protective mechanism, prevent increases in *P*_LEI_. Group A was characterized by an increase in end-inspiratory lung stress, as measured by the *P*_LEI_, with increasing *E*_lung_, whereas patients in Group B showed a decrease or no change in *P*_LEI_.

### Statistical analysis

Values are presented as median (interquartile range) or counts (percentage) unless otherwise stated. Normal distribution was checked by the Shapiro–Wilk test and comparisons within and between patients were performed by nonparametric or parametric tests, as appropriate. The diagnostic accuracy of Δ*P*_rs_ in detecting Δ*P*_lung_ ≥ 12 cmH_2_O was evaluated using the receiver operating characteristic (ROC) method [29, 30]. The effect of *E*_lung_ deciles on end-expiratory transpulmonary pressure (*P*_LEE_) and ΔPgas was analyzed using a linear mixed-effect model. A similar analysis was performed to examine the effect of ΔPgas on *P*_LEE_, as well as that of *P*_LEE_ on Δ*P*_lung_/Δ*P*_rs_. Regression analysis with curve estimation was performed on average values per decile between *E*_lung_ and *P*_LEE_, *E*_lung_ and ΔPgas, *P*_L__EE_ and Δ*P*_lung_/Δ*P*_rs_, and ΔPgas and *P*_LEE_ and the coefficient of determination (*r*^2^) was calculated. Patients were classified into Group A if, within each patient, there was a significant linear increase in *P*_LEI_ with increasing *E*_lung_. Binary logistic analysis was performed to examine if patients’ characteristics and outcomes can predict the pattern of response to changes in *E*_lung_. *P* < 0.05 was the statistically significant threshold. Statistical analysis was performed by using SPSS 26 software.

## Results

We obtained demographic, clinical, and ventilation data from 31 patients (22 instrumented with both esophageal and gastric balloons) during a 30-month period (Table 1). Data collected during 468 h of ventilation with PAV+ were examined and a total of 44,836 artifact-free occluded breaths were analyzed.

**Table 1:** Patients’ characteristics

Age (years)	68 (63–73)
Sex (M/F)	14/17
PBW, kg	61.4 (52.4–69.6)
BMI, kg/m^2^	31.6 (27.5–35.6)
COVID-19 status (Yes/No)	13/18
APACHE-II*	17.0 (14.0–19.5)
SOFA score*	8.0 (6.0–9.0)
PaO_2_/FIO_2_*	180 (153–210)
PaO_2_, mmHg*	85.0 (77.5–94.5)
PaCO_2_, mmHg*	38.0 (36.0–44.5)
pH	7.35 (7.29–7.40)
PEEP, cmH_2_O*	12 (9–15)
*V*_T_, ml/kg*	6.5 (5.9–7.3)
*E*_rs_, cmH_2_O/l*	25.0 (20.0–30.8)
*E*_lung_, cmH_2_O/l^‡^	20.1 (12.9–24.1)
*E*_cw_, cmH_2_O/l^‡^	7.8 (5.0–9.6)
Δ*P*_rs_, cmH_2_O*	10.0 (8.5–12.0)
Δ*P*_lung_, cmH_2_O^‡^	7.5 (4.5–10.0)
Δ*P*_lung_/ΔP_rs_^‡^	0.74 (0.62–0.79)
Days on MV at inclusion	7.0 (4.5–9.5)
Days on MV after inclusion	6.0 (3.5–14.5)
Total days on MV	14.0 (8.5–24.5)
ICU LOS (days)	20.0 (13.5–29.0)
ICU mortality, %	19.4

Values are median (1st to 3rd quartiles) or counts (percentage). PBW; Predicted body. BMI; Body mass index. APACHE-II; Acute Physiology and Chronic Health Evaluation II. SOFA; Sequential organ failure assessment. PaO_2_, PaCO_2_; Partial pressure of arterial O_2_ and CO_2_, respectively. PEEP; Positive end-expiratory pressure. *V*_T_; Tidal volume. *E*_rs_, *E*_lung_, *E*_cw_; Elastance of respiratory system, lung and chest wall, respectively. Δ*P*_rs_; driving pressure of respiratory system. Δ*P*_lung_; driving transpulmonary pressure. MV; Mechanical ventilation. ICU; Intensive care unit. LOS; Length of stay

*Data on Day 1 of control mechanical ventilation (passive, *n* = 31)

^‡^Data during control mechanical ventilation (passive) before switching to BiPAP or PAV+ (*n* = 15)

The results of 7-brMA analysis and analysis of all occluded breaths were similar, except at high values of Δ*P*_lung_ where 7-brMA analysis eliminated the sporadic high values (Additional file 2: Figs. S2, S3). Details of recorded parameters on the day of the study and the variation of Δ*P*_lung_ and other respiratory variables during the recording period are shown in Additional file 2: Tables S1 and S2.

### Primary outcomes

#### Occurrence of injurious lung stress

**Fig. 2:**
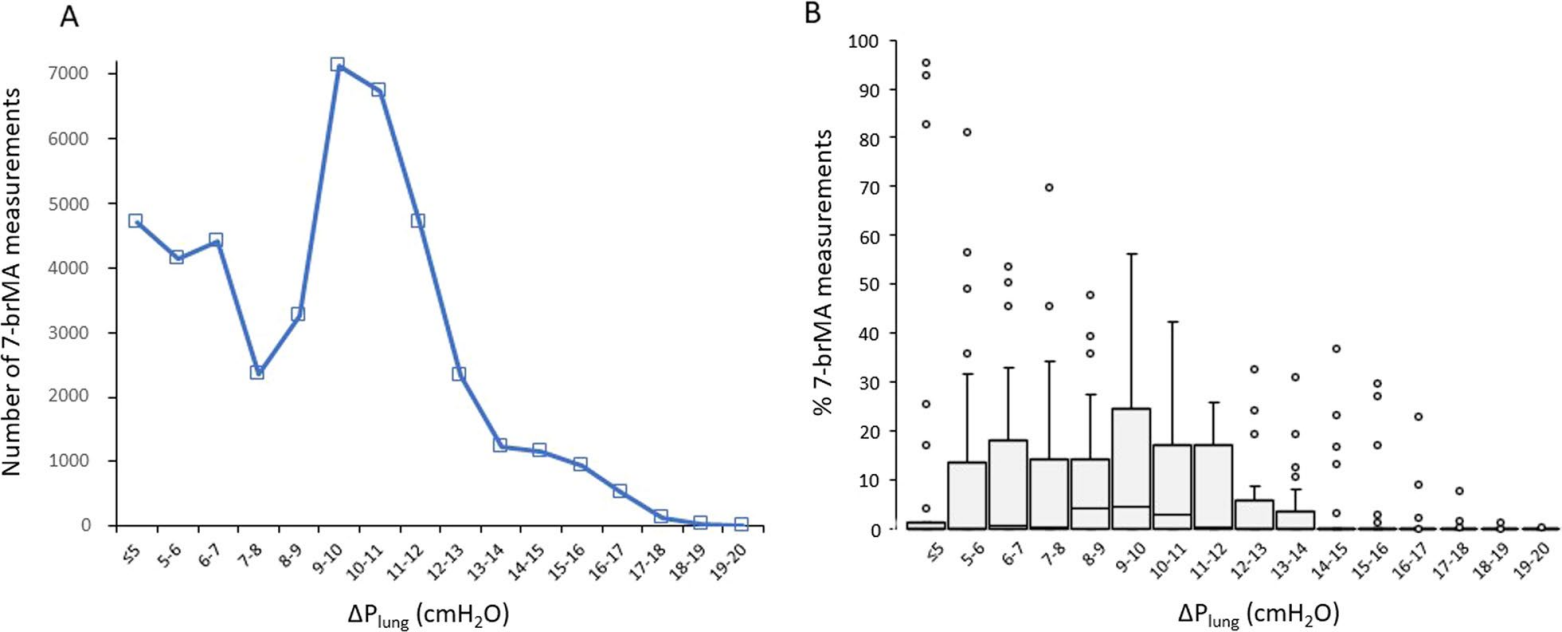
Number of 7-breath moving average measurements (**A**) and % of total measurements (**B**) with Δ*P*_lung_ within the range of each cmH_2_O from ≤ 5 cmH_2_O to maximum values. Outliers are shown by circles

The median number of 7-brMA measurements and the percentage of these measurements where Δ*P*_lung_ aligns within the range of each cmH_2_O, from ≤ 5 cmH_2_O to the maximum value is illustrated in Fig. 2. Δ*P*_lung_ values ≥ 12 cmH_2_O were observed in 15 out of 31 patients (Additional file 3: Individual data). One patient had constantly Δ*P*_lung_ ≥ 12 cmH_2_O, while in the remaining 14 patients, Δ*P*_lung_ above and below this threshold were noted. The median (IQR) percentage of measurements with Δ*P*_rs_ < 15 cmH_2_O and Δ*P*_lung_ < 12 cmH_2_O is presented in Table 2.

**Table 2:** Percentage of 7-brMA measurements with quasi-static and dynamic lung stress and respiratory effort indices within optimum range

Optimum range	(% of total 7-brMA measurements)
Δ*P*_rs_ < 15 cmH_2_O	100 (99.1–100)
Δ*P*_lung_ < 12 cmH_2_O	100 (88.0–100)
Plung_sw_ < 15 cmH_2_O	85.0 (33.1–100)
Plung_sw_ < 20 cmH_2_O	100 (97.1–100)
3 ≤ ΔPdi < 12 cmH_2_O*	98.0 (83.3–100)
3 ≤ Pmus_sw_ < 15 cmH_2_O^‡^	89.2 (49.3–100)

Values are median and interquartile range (IQR). Δ*P*_rs_; driving pressure of respiratory system. Δ*P*_lung_; driving transpulmonary pressure. ΔPdi; transdiaphragmatic pressure swings. Plung_sw_; dynamic transpulmonary pressure swings. Pmus_sw_; Respiratory muscles (inspiratory and expiratory) pressure swings

*In 3 patients ΔPdi < 3 cmH_2_O was observed for 11.9%, 35.4%, and 21.2% of total measurements

^‡^Pmus_sw_ < 3 cmH_2_O was not observed

The number and percentage of 7-brMA measurements where Δ*P*_rs_ aligns within the range of each cmH_2_O, from ≤ 5 cmH_2_O to the maximum value is illustrated in Additional file 2: Figure S4.

#### Relationship between Δ*P*_rs_ and Δ*P*_lung_

**Fig. 3:**
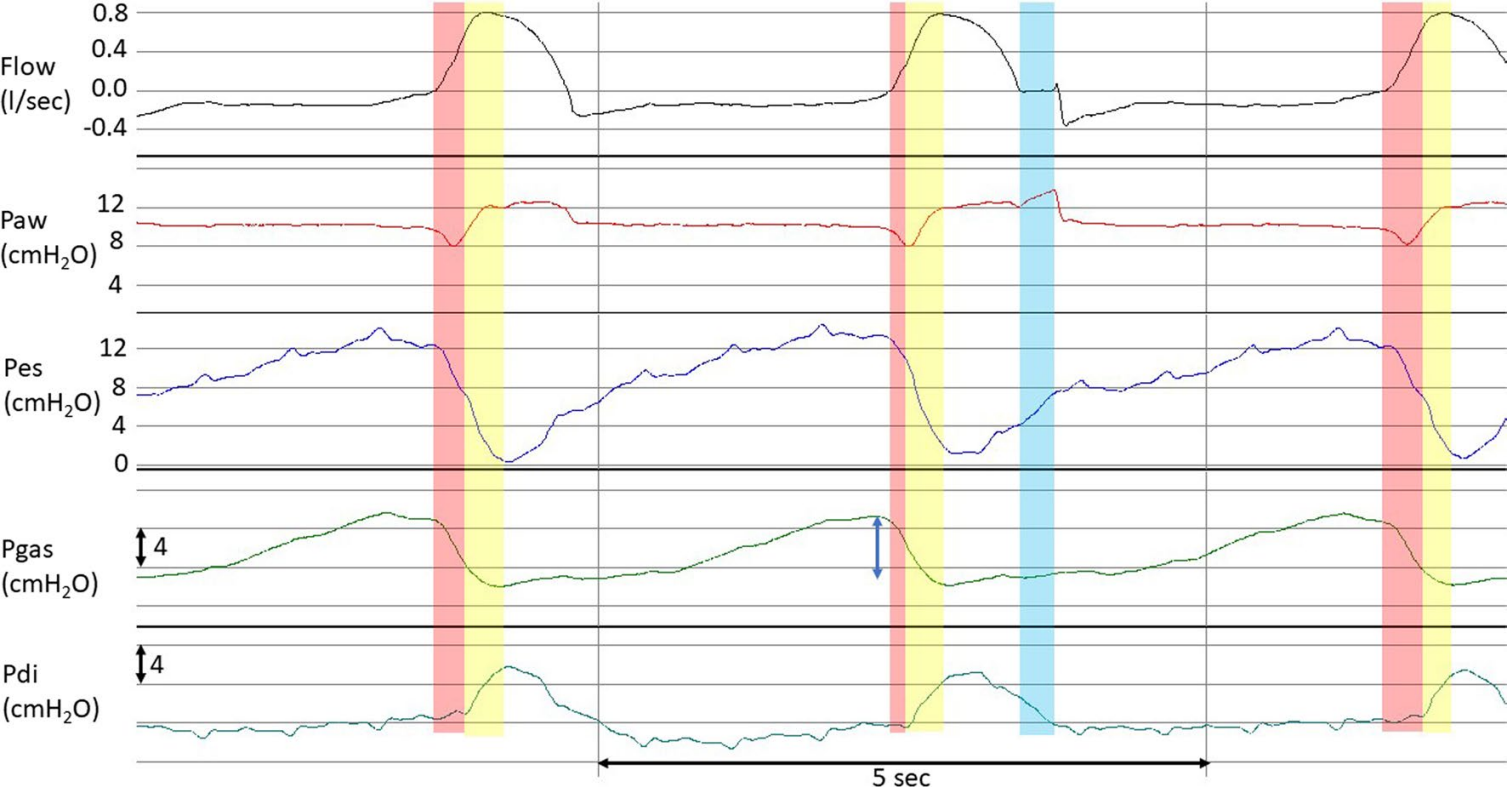
Flow and airway, esophageal, gastric, and transdiaphragmatic pressures in a patient with ARDS ventilated on PAV+ . An occluded and two, preceding and following, un-occluded breaths are shown. Notice that in all breaths inspiratory flow initially is generated only be relaxation of expiratory muscles (red areas). Thereafter, the diaphragm contracts, while expiratory muscles continue to relax (yellow areas). At the beginning of inflation of the occluded breath (zero flow) *P*_LEE_ is −3.44 cmH_2_O and at the end of occlusion (end of blue area) *P*_LEI_ is 6.48 cmH_2_O. The calculated Δ*P*_lung_ is 9.92 cmH_2_O. The corresponding values of Paw are 9.62 and 13.64 cmH_2_O and Δ*P*_rs_ is 4.02 cmH_2_O. Totally passive inspired volumes (integrated flow-time red area) in these three breaths are 76, 28 and 85 ml, respectively. The end of relaxation of expiratory muscles occurred when inspired volumes (sum of red and yellow areas) were 265 ml (1st breath), 247 ml (2nd breath), and 268 ml (3rd breath). Notice that before the occluded breath gastric pressure increased by 6.4 cmH_2_O (blue double edge arrow), indicating significant expiratory muscle activity that is able to decrease expiratory volume below that determined by PEEP. Notice also that the drop in Pgas due to expiratory muscle relaxation was 7.0 cmH_2_O. Observe also that at the end of occlusion Pdi returned to baseline and during occlusion the change in Pgas was negligible (0.3 cmH_2_O), indicating passive condition during measurements of *P*_plat_. Tidal volume of occluded breath was 562 ml and calculated elastance of respiratory system was 7.2 cmH_2_O/l, while that of the lung 17.8 cmH_2_O. Δ*P*_lung_/ΔP_rs_ (and *E*_lung_/*E*_rs_) was 2.5

Twenty-one patients exhibited significant expiratory muscle activity (16 had average ΔPgas > 1 cmH_2_O and 5 exhibited signs of active expiration in V′ and Pes waveforms). In several of them, expiratory muscle relaxation contributed to a portion of the *V*_T_ measured (Fig. 3). This led to an underestimation of Δ*P*_lung_ by Δ*P*_rs_. Most patients (24/31, 77.4%) had readings of Δ*P*_lung_ that exceeded Δ*P*_rs_ due to this underestimation. The median number of such measurements was 332 (13–490), accounting for 31.7% (2.2–94.5%) of the total measurements. In two patients, Δ*P*_lung_ always exceeded Δ*P*_rs_.

**Fig. 4:**
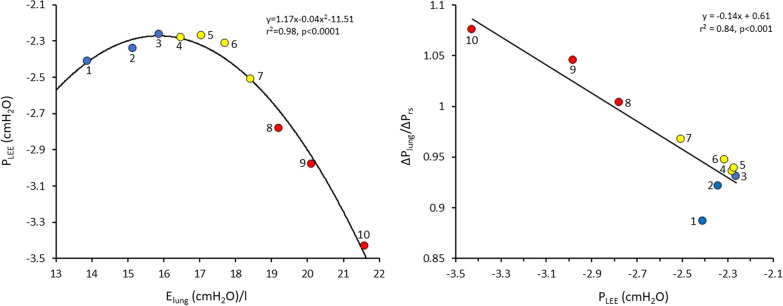
Relationship between lung elastance (*E*_lung_) and transpulmonary pressure at the end of expiration (*P*_LEE_) (Left) and *P*_LEE_ and ratio of driving transpulmonary pressure to that of respiratory system (Δ*P*_lung_ /ΔP_rs_) (Right). Each circle represents the average values of these variables in each of the 10 segments characterized by increasing *E*_lung_. Blue circles: Deciles 1–3 (low *E*_lung_). Yellow circles: Deciles 4–7 (moderate *E*_lung_). Red circles: Deciles 8–10 (high *E*_lung_). Notice that the highest *E*_lung_ (Decile 10) is associated with the lowest *P*_LEE_ and the highest Δ*P*_lung_/Δ*P*_rs_. Observe also that at highest *E*_lung_ (Decile 10) average Δ*P*_lung_ is greater than Δ*P*_rs_. The number in each circle indicates the corresponding decile. Notice that *P*_LEE_ begins to decrease after decile 6. This is reflected in almost constant Δ*P*_lung_/Δ*P*_rs_ from decile 1 to 6

A total of 310 deciles with progressive increases in *E*_lung_ were analyzed (10 deciles per patient). When *E*_lung_ increased, Δ*P*_lung_ increased in all patients (Additional file 2: Table S3). There was a highly significant relationship of quadratic function (*y* = *a* + *b*1*x* + *b*2*x*^2^) between per decile average values of *E*_lung_ and *P*_LEE_ and a negative linear relationship of *P*_LEE_ and Δ*P*_lung_/Δ*P*_rs_ (Fig. 4). The decrease in *P*_LEE_ with increasing *E*_lung_ was due to expiratory muscle contraction, as reflected by a quadratic function relationship between *E*_lung_ and Δ*P*_gas_ (Additional File 2: Fig. S5).

#### Response to increasing Elung by patient Group

**Fig. 5:**
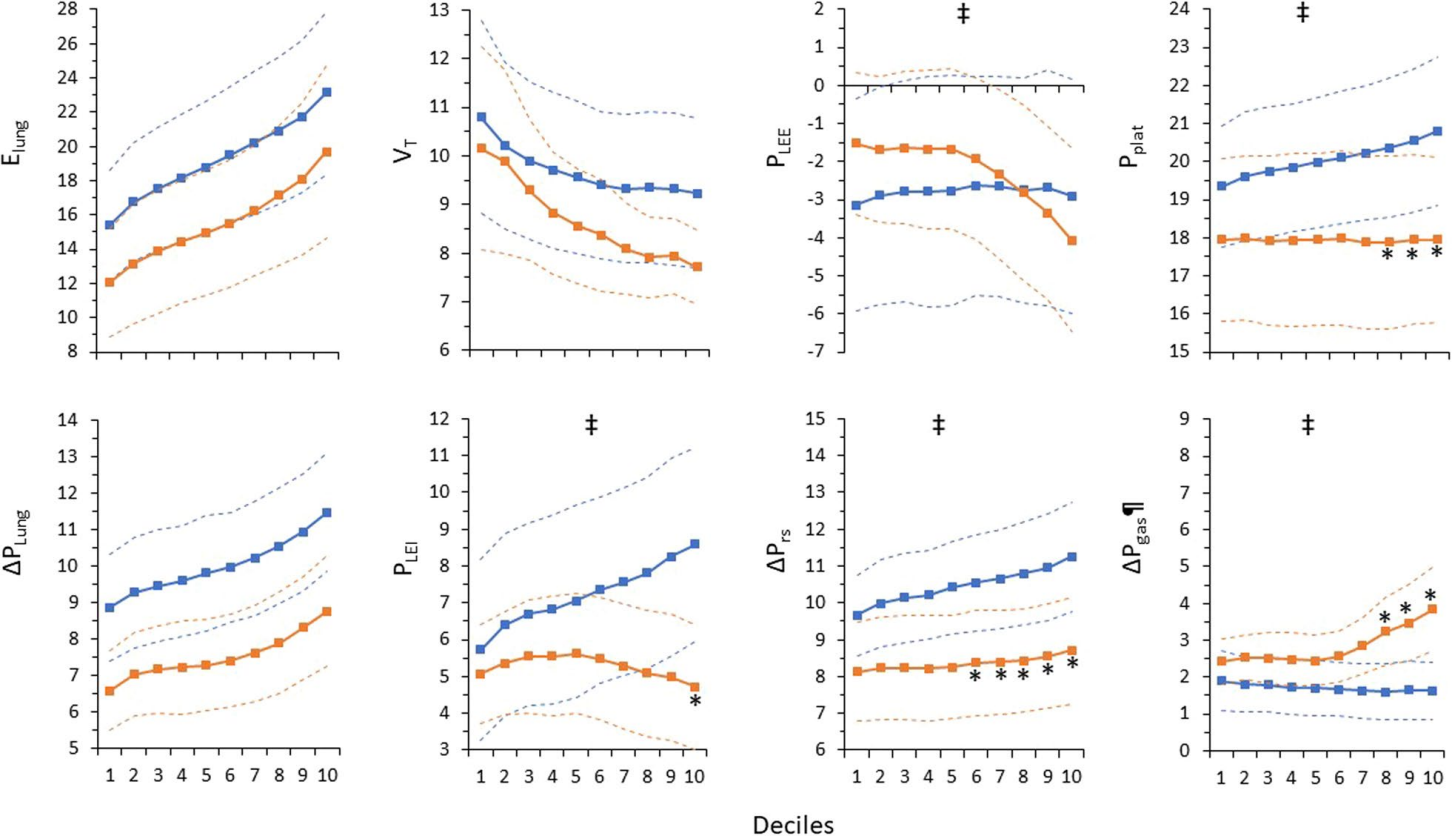
Effects of a progressive increase in *E*_lung_ (Decile 1: the lowest *E*_lung_; Decile 10 the highest *E*_lung_) on average respiratory variables in Group A (blue squares connected by blue lines, characterized by a linear increase in *P*_LEI_ with increasing *E*_lung_) and Group B (orange squares connected by orange lines, characterized by no increase in *P*_LEI_ with increasing *E*_lung_). Blue and orange dashed lines indicate standard deviation range in Groups A and B, respectively. Notice the significant interaction between groups in transpulmonary pressure at end-inspiration (*P*_LEI_) and end-expiration (*P*_LEE_), driving pressure (Δ*P*_rs_), end-inspiratory plateau pressure (*P*_plat_), and gastric pressure increase during expiration (Δ*P*_gas_). ‡Significant interaction between Groups (Split-plot ANOVA). *Significant difference from the corresponding value of Group A. ¶Pertains to 22 patients (11 in each group)

Seventeen out of thirty-one patients were included in Group A and the remaining 14 were in Group B. Although with increasing *E*_lung_, Δ*P*_lung_ increased similarly between groups, in Group A this increase was mainly due to a *P*_LEI_ increase, while in Group B to a *P*_LEE_ decrease. With increasing *E*_lung_, contrary to Group A, Group B was characterized by constant Δ*P*_rs_ and *P*_plat_, a significant decrease in *P*_LEE_, and an increase in Δ*P*_gas_ (Fig. 5). The response of other variables is shown in Additional file 2: Table S4. Similar results were observed when only patients with gastric pressure measurements (*n* = 22) were analyzed (Additional file 2: Fig. S6). The linear mixed-effects model analysis, with *P*_LEE_ as the dependent variable, *E*_lung_ deciles and group category as fixed effects, and each subject as a random effect, demonstrated a significant effect (*P* < 0.001) of *E*_lung_ on *P*_LEE_. There was no effect of group category on *P*_LEE_. Similarly, a significant effect of Δ*P*_gas_ on *P*_LEE_ was also observed. When Δ*P*_lung_/Δ*P*_rs_ was used as the dependent variable, there was a significant effect (*P* < 0.001) of *P*_LEE_, as a fixed variable, but there was no significant effect of group category. Binary logistic regression showed that none of the patients’ characteristics, including age and body mass index, length of ICU stay, days on mechanical ventilation, and ICU outcome, predicted the Group classification.

#### Accuracy of Δ*P*_rs_ to predict injurious Δ*P*_lung_

**Fig. 6:**
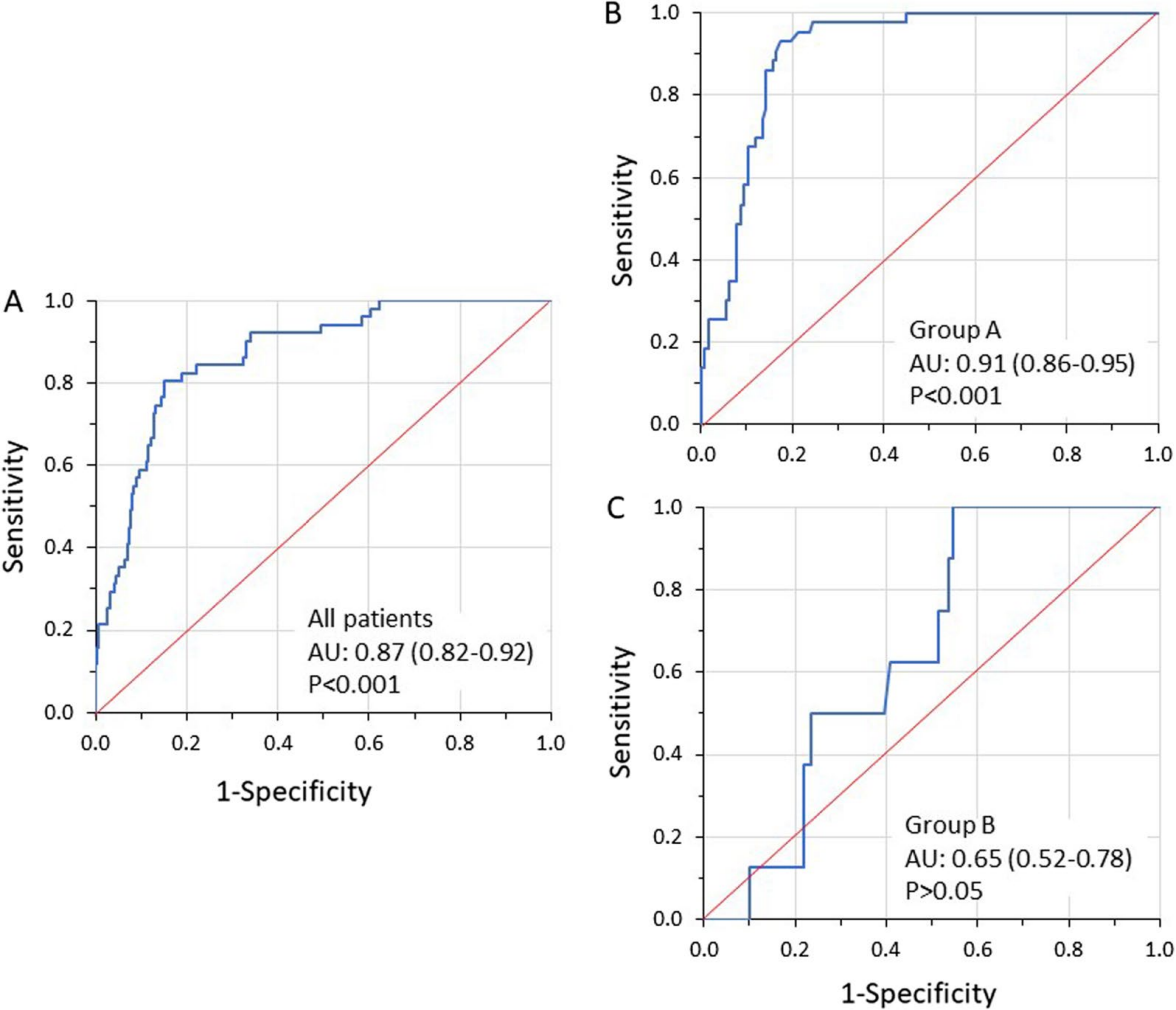
Receiver operating characteristics curves (blue lines). Area under the curve (AUC) of driving pressure of respiratory system (Δ*P*_rs_) to predict transpulmonary driving pressure (Δ*P*_lung_) ≥ 12 cmH_2_O in all patients (**A**, 310 segments) and patients of Group A (**B**, 170 segments) and Group B (**C**, 140 segments). Notice that contrary to patients of Group A, in patients of Group B Δ*P*_rs_ does not have a significant predictive value for Δ*P*_lung_ ≥ 12 cmH_2_O. Values of AUC are with 95% confidence intervals, and *P* values pertain to the test of AUC to the guess. Best cutoff measurements based on Youden index was 11.5 cmH_2_O in all patients (**A**) and 11.8 cmH_2_O in patients of Group A (**B**)

ROC curve analysis revealed that, although Δ*P*_rs_ had high accuracy for detecting Δ*P*_lung_ ≥ 12 cmH_2_O in the overall population, this effect was due to patients of Group A. In Group B, Δ*P*_rs_ showed no predictive capacity for detecting injurious Δ*P*_lung_ (Fig. 6).

### Secondary outcomes

#### Dynamic transpulmonary pressure swings and effort

The median (IQR) percentage of measurements with Plung_sw_, inspiratory Pdi swings (ΔPdi), and Pmus_sw_ falling within a range considered optimum [19, 21, 22, 31] is presented in Table 2.

Δ*P*_lung_ ≥ 12 cmH_2_O was associated with higher values of effort indices, *V*_T_, and Plung_sw_ (Additional file 2: Table S5). As *E*_lung_ increased, Plung_sw_ and efforts indices significantly increased, despite significant decreases in *V*_T_ (Additional file 2: Table S3).

## Discussion

In this study, tidal lung stress was documented in ARDS patients during their early transmission from controlled mechanical ventilation to assisted breathing with PAV+. The main findings are as follows: (1) Half of the patients (51.6%) did not exhibit Δ*P*_lung_ exceeding 12 cmH_2_O and in cases where it was observed, such instances were of limited duration. (2) Most of the time, Plung_sw_ and inspiratory effort indices were within a range considered optimum. (3) A significant proportion of patients exhibited expiratory muscle recruitment and a reduction in end-expiratory lung volume, as evidenced by decreased *P*_LEE_. (4) In these patients, the relaxation of expiratory muscles contributed to *V*_T_ and as a result, Δ*P*_rs_ underestimated Δ*P*_lung_, making it non-suitable as an alternative for tidal static lung stress.

Certain methodological issues of the study should be discussed first. The calculation of Δ*P*_rs_ during PAV+ ventilation, relies on the measurement of *P*_plat_, by random application of short end-inspiratory occlusions. Younes et al. have shown that since with PAV+ there is a link between the end of neural and mechanical inflation, this method provides a reliable estimate of passive elastic recoil pressure of the respiratory system at the corresponding *V*_T_ [10]. Indeed, we observed that Pdi at the end of occlusion had returned to baseline and in the vast majority of the patients, Pgas remained constant during the pause time, assuring passive condition (Fig. 3). In a few patients, a small increase in Pgas (0.5–< 1.5 cmH_2_O) was occasionally observed, leading to an overestimation of the measured *P*_plat_ and Δ*P*_rs_ by this amount. This, however, did not affect the computation of the *P*_LEI_, since expiratory muscle contraction during occlusion equally elevates Paw and Pes. Secondly, consistent with earlier investigations [32–34], *P*_LEE_ remained predominantly negative throughout the recording period in 20 out of 31 patients. While this observation might raise concerns about the precision of Pes measurements [35, 36], a recent study involving lung-injured pigs and human cadavers assessed directly pleural pressure and demonstrated that Pes accurately mirrors pleural pressure in lung regions proximal to the esophageal balloon [37]. In this study, consistently negative *P*_LEE_ values were observed, whether based on pleural or esophageal pressure measurements.

### Transpulmonary driving pressure, dynamic transpulmonary pressure swings, and effort indices

It has been demonstrated that keeping Δ*P*_lung_ < 12 cmH_2_O and Plung_sw_ < 20 cmH_2_O in patients with ARDS without spontaneous breathing activity is linked to improved survival [18, 38]. These thresholds have been also suggested as targets during assisted breathing [22]. We demonstrated that Δ*P*_lung_ ≥ 12 cmH_2_O occurred rarely and for a short period of time, while in half of the patients (51.6%) such values were never observed (Table 2). Similarly, Plung_sw_ remained within the safe range for most of the time, even when the more conservative threshold of 15 cmH_2_O was examined. However, it is unknown if these results, documented during PAV+ ventilation, are also applicable in conventional assisted modes. Proportional ventilation, including PAV+ and neurally adjusted ventilator assist (NAVA), allows control of breathing system to regulate *V*_T_ using chemical and reflex feedback mechanisms [39, 40], that tend to naturally protect the lung from over-distension [41, 42].

While direct studies in humans are lacking, it is generally considered safe to maintain ΔPdi within the range of 3 to 12 cmH_2_O and Pmus_sw_ within the range of 3 to 15 cmH_2_O to prevent both over-assistance and under-assistance, thereby ensuring the protection of the lungs and diaphragm [21]. In our study, primary physicians, who did not have access to study data, selected a level of assistance that averaged 50%. At this average assist, which amplifies inspiratory muscle pressure by a factor of 2 [43], both ΔPdi and, to a lesser extent, Pmus_sw_ fell within the optimal ranges.

These results are in contrast to those obtained by Di Mussi et al. [44]. In their study, 16 patients transitioned from control to pressure support ventilation, with continuous monitoring of electrical activity of the diaphragm (EAdi) over a 12-h period. They observed that 50% of breaths were either over-assisted (28%) or under-assisted (22%). Notwithstanding that in the study of Di Mussi et al. [44] EAdi was used as an index of under- or over-assistance, this disparity can be attributed to the functional principles of pressure support, which, unlike PAV+, hinders the control of breathing system in regulating *V*_T_ [45, 46]. The observed greater variation in Pmus_sw_ in our study (Table 2) is likely influenced by expiratory and accessory inspiratory muscle pressures, which contribute to the calculation of Pmus, as well as uncertainties related to passive chest wall properties.

### Driving pressure of respiratory system and relationship to driving transpulmonary pressure

Consistent with our previous studies involving a general population of critically ill patients [15, 17], the current study showed that in ARDS patients ventilated with PAV+, Δ*P*_rs_ rarely exceeded 15 cmH_2_O. Although in our previous studies, we postulated that Δ*P*_rs_ < 15 cmH_2_O was associated with acceptable tidal lung stress, the current investigation challenges this assumption. We found a considerably low (11.5 cmH_2_O) threshold of Δ*P*_rs_ for detecting Δ*P*_lung_ ≥ 12 cmH_2_O (Fig. 6), suggesting that the calculated Δ*P*_rs_ underestimated Δ*P*_lung_. These results conflict those reported recently by Perez et al. in a small ARDS patients’ cohort during pressure support ventilation [47]. In that study, Δ*P*_rs_ had an excellent precision to predict Δ*P*_lung_, with a value of 15 cmH_2_O being identified as the best threshold for detecting Δ*P*_lung_ ≥ 12 cmH_2_O. However, Perez et al. selectively analyzed only a few occluded breaths while patients with expiratory muscles activity were excluded. The vast majority of our patients exhibited expiratory muscle activity during expiration, limiting the applicability of the findings of Perez et al. in routine clinical practice.

The explanation why Δ*P*_rs_ underestimated Δ*P*_lung_, as well as why in several measurements Δ*P*_lung_ was found higher than Δ*P*_rs_ lies in the effect of end-expiratory lung volume on Δ*P*_rs_ calculation (Fig. 1). When analyzing all breaths, a negative linear relationship between *P*_LEE_ and Δ*P*_lung_/Δ*P*_rs_ was observed in 81% of patients. Additionally, by analyzing the response to *E*_lung_ changes, the linear mixed-effect model analysis found a significant effect of ΔPgas on *P*_LEE_, as well as of *P*_LEE_ on Δ*P*_lung_/Δ*P*_rs_. These findings indicate that in several patients, expiratory muscle contraction reduced end-expiratory lung volume, as reflected by a lower *P*_LEE_. In these patients, Δ*P*_rs_ underestimated the true driving pressure of the respiratory system because it assumed that the elastic recoil pressure when volume started to enter the lungs was equal to PEEP. However, expiratory muscle activation had decreased lung volume to a lower value than that corresponding to PEEP, and the very first moment that expiratory muscles relaxed, volume started to enter the lungs as a result of an alveolar pressure lower than PEEP (Fig. 1 and 3). Obviously, underestimation of the actual respiratory system driving pressure means underestimation of the calculated respiratory system elastance. Additionally, at lung volumes well below the level determined by PEEP, not only is the actual change in Δ*P*_rs_ higher than the calculated value, but the elastance of the respiratory system may also increase. In this situation, the actual Δ*P*_rs_ is even higher than what is calculated assuming a linear relationship between pressure and volume during lung inflation.

### Response of respiratory variables to Elung changes

As anticipated, *E*_lung_ changed considerably in all patients over the observation period and Δ*P*_lung_ unanimously increased at higher *E*_lung_. However, we found two distinct responses to *E*_lung_ increases. One group of patients (Group A), responded with increased *P*_LEI_, which drove the increase in Δ*P*_lung_. The second group (Group B), maintained constant or even decreased the end-inspiratory lung stress (*P*_LEI_) at higher *E*_lung_, and the higher Δ*P*_lung_ resulted from *P*_LEE_ decrease. Gastric pressure measurements showed that the observed decrease in *P*_LEE_ was accomplished by a considerable increase in expiratory muscle activity. It is of interest to note that the further decrease in *P*_LEE_ and increase in Δ*P*_gas_ occurred when *E*_lung_ increased by 27% (Fig. 4 and Additional file 2: Fig. S5), signifying a considerable increase in ventilatory demands. This response played a pivotal role in effectively mitigating the extent of end-inspiratory lung stress associated with a specific magnitude of Δ*P*_lung_ elevation. It is not clear why this different response was observed. However, in Group A, the consistently higher *E*_lung_ at all deciles and the already low *P*_LEE_ values at low *E*_lung_ (Fig. 5) suggest that some patients could not further decrease their already low-end-expiratory lung volume. Nevertheless, the response pattern was not dependent on patients’ characteristics, total duration of mechanical ventilation, length of ICU stay, and ICU outcome.

Interestingly, in Group B patients, Δ*P*_rs_ remained constant and, consequently, lacked predictive value for high Δ*P*_lung_. This underscores the importance of end-expiratory lung volume reduction below that corresponding to PEEP, as the primary determinant of Δ*P*_lung_/Δ*P*_rs_. Therefore, interpreting Δ*P*_rs_ as an index of tidal lung stress should be approached with great caution. Although the Δ*P*_rs_-Δ*P*_lung_ relationship was examined during PAV+, similar results should be expected during all modes of assisted mechanical ventilation, since the underestimation of Δ*P*_lung_ by Δ*P*_rs_ does not depend on the mode but on the ability of expiratory muscles to decrease end-expiratory lung volume below that determined by PEEP.

We cannot determine whether the distinct response to deteriorating lung elastance conferred a lung-protected benefit in one group compared to the other. Lower *P*_LEE_ was associated with minimal or no increase in end-inspiratory lung stress despite Δ*P*_lung_ increase (Fig. 5). However, it remains uncertain to what extent this provides protection, as decreases in end-expiratory lung volume may potentially be associated with lung injury (atelectrauma), derecruitment, and gas exchange abnormalities [48].

### Limitations

This study has certain limitations that should be considered. Firstly, end-expiratory lung volume changes were not directly monitored; instead, *P*_LEE_ was utilized for this purpose. However, we believe that *P*_LEE_ can provide valuable insights into the direction of change. When *E*_lung_ remains constant or increases, a decrease in *P*_LEE_ is indicative of a reduction in end-expiratory lung volume [11]. Therefore, we feel confident that in our study, a decrease in *P*_LEE_ resulted from lower end-expiratory lung volume. Secondly, this single-center study included a group of patients with ARDS, who were enrolled when the primary physician opted for PAV+ as the initial assisted mode, following judgment of safety for allowing spontaneous breathing activity. Thus, the time lag between intubation and assisted ventilation differed. However, based on clinical judgment, the patients were included at relatively early stages of recovery from ARDS, when the respiratory drive was relatively high, as evidenced by the significant proportion displaying expiratory muscle activity [45]. Nevertheless, these findings may not be generalizable to all critically ill patients, although this patient group is particularly relevant when assessing Δ*P*_rs_ as a surrogate for tidal lung stress. Thirdly, this prospective observational study is subject to the inherent biases associated with patient selection and the lack of strict adherence to specific algorithms when titrating PEEP and the level of assist with PAV+ [49]. Nevertheless, this can also be considered a strength, since it allows us to capture the impact of every day clinical practice on Δ*P*_lung_ and effort indices. Fourthly, since this was beyond the scope of the study, the impact of factors that influence the recruitment of expiratory muscles, such as respiratory acidosis, sedation, and diaphragmatic weakness on group response could not be assessed. Finally, the pendelluft phenomenon, which may occur in patients with high respiratory drive and unpredictably change tidal volume, was not considered.

## Conclusions

Transpulmonary driving pressures and inspiratory efforts were largely maintained within a safe range during proportional assist ventilation. Contrary to existing assumptions, the respiratory system driving pressure underestimated the transpulmonary driving pressure due to expiratory muscle activity which lowers end-expiratory lung volume below that determined by PEEP. This phenomenon, which should occur regardless of the mode of support, limits the usefulness of respiratory system driving pressure as a substitute for transpulmonary driving pressure in patients with active breathing.

## Supplementary Information

**Additional file 1.** Figure S1.

**Additional file 2.** Supplementary Methods, Results, Figures, Tables and References.

**Additional file 3.** Individual data of Transpulmonary driving pressure over time.

## Abbreviations

Δ*P*_lung_: Transpulmonary driving pressure
Δ*P*_rs_: Driving pressure of the respiratory system
*P*_plat_: End-inspiratory plateau pressure
PEEP: Positive end-expiratory pressure
*V*_T_: Tidal volume
*P*lung_sw_: Transpulmonary pressure swings
ARDS: Acute respiratory distress syndrome
*V*′: Airflow
Paw: Airway pressure
Pes: Esophageal pressure
Pgas: Gastric pressure
*P*_lung _ (Paw-Pes): Transpulmonary pressure
Pdi (Pgas-Pes): Transdiaphragmatic pressure
Pmus_sw_: Respiratory muscles (inspiratory and expiratory) pressure swings
Δ*P*_gas_: Rise in gastric pressure during the expiratory phase
PEEPi: Dynamic intrinsic PEEP
7-brMA: Seven-breath moving average
*E*_lung_: Lung elastance
ROC: Receiver operating characteristic
AUC: Area under the receiver operating characteristic curve
ΔPdi: Inspiratory Pdi swings
*P*_LEI_: End-inspiratory transpulmonary pressure
*P*_LEE_: End-expiratory transpulmonary pressure
CMV: Control mechanical ventilation
PAV+: Proportional assist ventilation with load adjustable gain factors
ICU: Intensive care unit
