# Supplementary material for: Driving pressure of respiratory system and lung stress in mechanically ventilated patients with active breathing
Source: Crit Care. 2024 Jan 12;28:19. doi: 10.1186/s13054-024-04797-3 (PMC10785492; doi:10.1186/s13054-024-04797-3)
Supplement: Supplementary file 1 — Additional file 1. Figure S1. [file 13054_2024_4797_MOESM1_ESM.docx]

**Additional file 1**

**Driving pressure of respiratory system and lung stress**

**in mechanically ventilated patients with active breathing**

**Figures S1A and S1B.** Simplified model analysis showing the effect of decreasing end-expiratory lung volume below (V_EE<FRC_) that corresponding to PEEP (V_EE,PEEP_) on calculation of driving pressure of respiratory system (ΔP_rs_) and transpulmonary driving pressure (ΔP_lung_). For simplicity: a) elastance of lung (E_lung_) and chest wall (E_cw_) were kept constant at all lung volumes, b) total airway closure (and thus airway re-opening pressure) is absent and c) pendelluft does not occur.

In this model set and derived values are:

**Set values**

1. PEEP
2. Tidal volume (V_T_)
3. Lung volume below that determined by PEEP (V_EE<FRC_)
4. Pleural pressure (P_pl_) at the end of expiration in Fig. S1A (passive expiration)
5. Plateau pressure (P_plat_) in Fig. S1A
6. E_cw_ (20% of respiratory system elastance)
7. Expiratory muscles pressure at end-expiration (Pmus_exp_)

**Derived values**

1. Alveolar pressure (P_alv_) at the end of expiration and inspiration
2. P_pl_ at the end of expiration in the presence of expiratory muscle activity
3. P_pl_ at the end of inspiration
4. P_plat_ in Fig. S1C
5. Lung elastic recoil pressure (Transpulmonary pressure, P_lung_)
6. Chest wall elastic recoil pressure (P_cw_)
7. Respiratory system elastance (E_rs_)
8. E_lung_
9. ΔP_rs_
10. ΔP_lung_
11. ΔP_lung_/ΔP_rs_

**S1A:** Pressures (cmH_2_O) applied to respiratory system at the end of expiration and inspiration at zero flow in the absence of respiratory muscle activity (inspiratory and expiratory) at these volumes. Expiration is assumed to be passive. Transdiaphragmatic activity (Pdi) starts at V_EE,PEEP_.


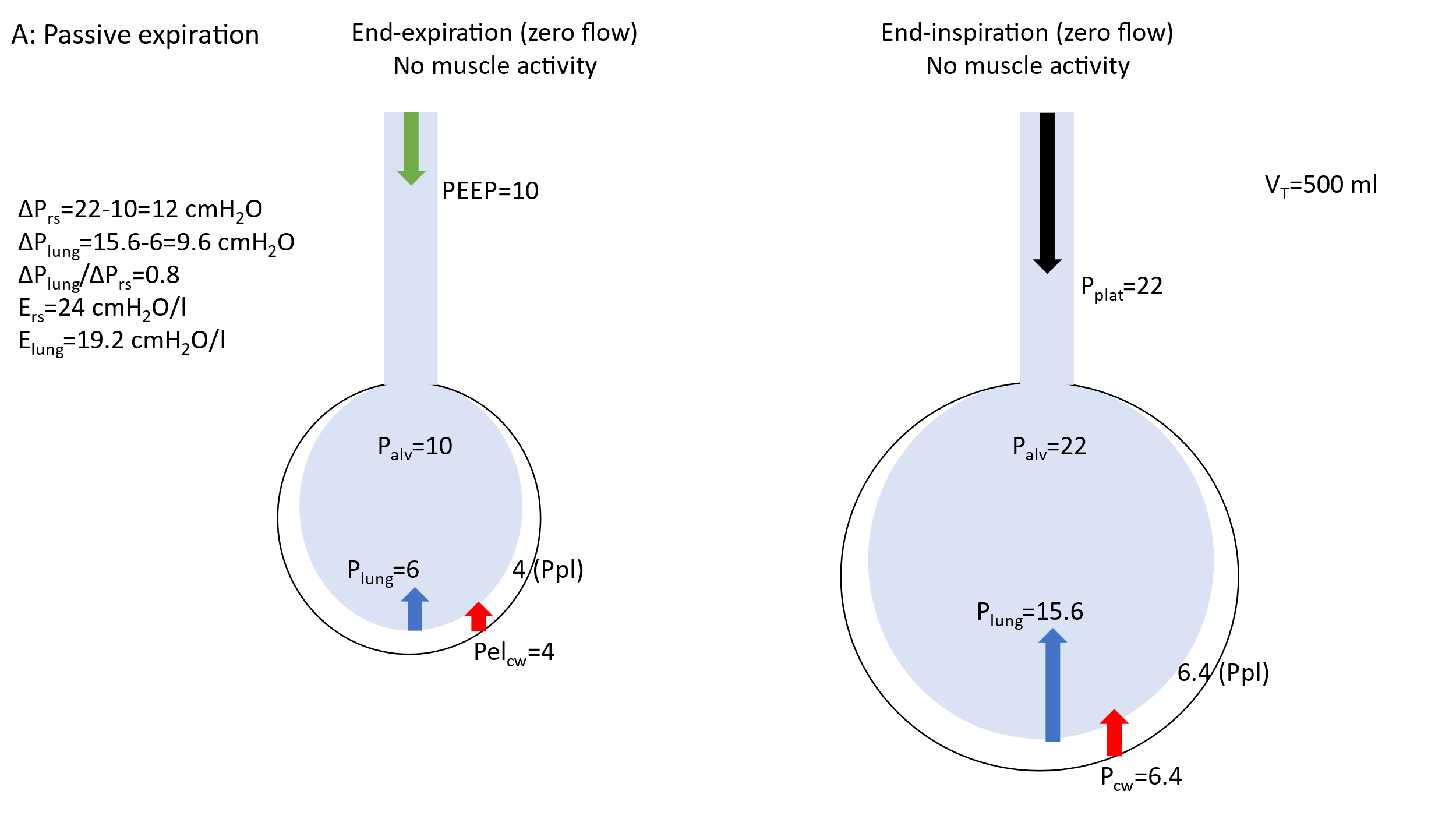


**Calculation of ΔPrs and Ers**

At end-expiration since flow is zero, PEEP (green arrow) and P_alv_ are equal. With Proportional assist ventilation (PAV+) at the end of brief end-inspiratory occlusion there is no respiratory muscle activity (see additional file 2). P_plat_ at the end of inspiratory occlusion (zero flow) is set to 22 cmH_2_O and is equal to P_alv_. Tidal volume (V_T_) is set to 500 ml. ΔP_rs_ is 12 cmH_2_O (P_plat_-PEEP=22-10) and E_rs_ is calculated as ΔP_rs_/V_T_=(22-10)/0.5=24 cmH_2_O/l.

**Calculation of ΔPlung, Elung and Ecw.**

P_pl_ at the end of expiration is set to 4 cmH_2_O and because expiration is passive end-expiratory lung volume is determined by PEPP (V_EE,PEEP_). Therefore, P_pl_ is the elastic recoil pressure of chest wall (P_cw_) at V_EE,PEEP_ (red arrow). Lung elastic recoil pressure (transpulmonary pressure, P_lung_) is 6 cmH_2_O (P_lung_=Palv-P_pl_). If E_cw_ is set to 20% of E_rs_, E_cw_=24x0.2=4.8 cmH_2_O/l. P_pl_ at end of inspiration (measured at the end of occlusion) is the sum of P_cw_ at V_EE,PEEP_ (4 cmH_2_O) plus the increase in P_cw_ due to volume increase above V_EE,PEEP_ (4.8x0.5=2.4 cmH_2_O). P_pl_=P_cw_=4+2.4=6.4 cmH_2_O. P_lung_ at end inspiration is P_alv_-P_pl_=22-6.4=15.6 cmH_2_O (blue arrow). Elastic recoil pressure of respiratory system at end-inspiration is 22 cmH_2_O (P_lung_+P_cw_). ΔP_lung_=15.6-6.0=9.6 cmH_2_O, E_lung_=9.6/0.5=19.2 cmH_2_O/l and ΔP_lung_/ΔP_rs_=0.8.

**S1B:** Pressures (cmH_2_O) applied to respiratory system at the end of expiration and inspiration at zero flow at presence of expiratory muscle activity during expiration (Same simulated patient of S1A). Diaphragmatic contraction begins when flow is expiratory and before the end of complete relaxation of expiratory muscle. Diaphragmatic activity (Pdi) is similar to Fig. S1A.


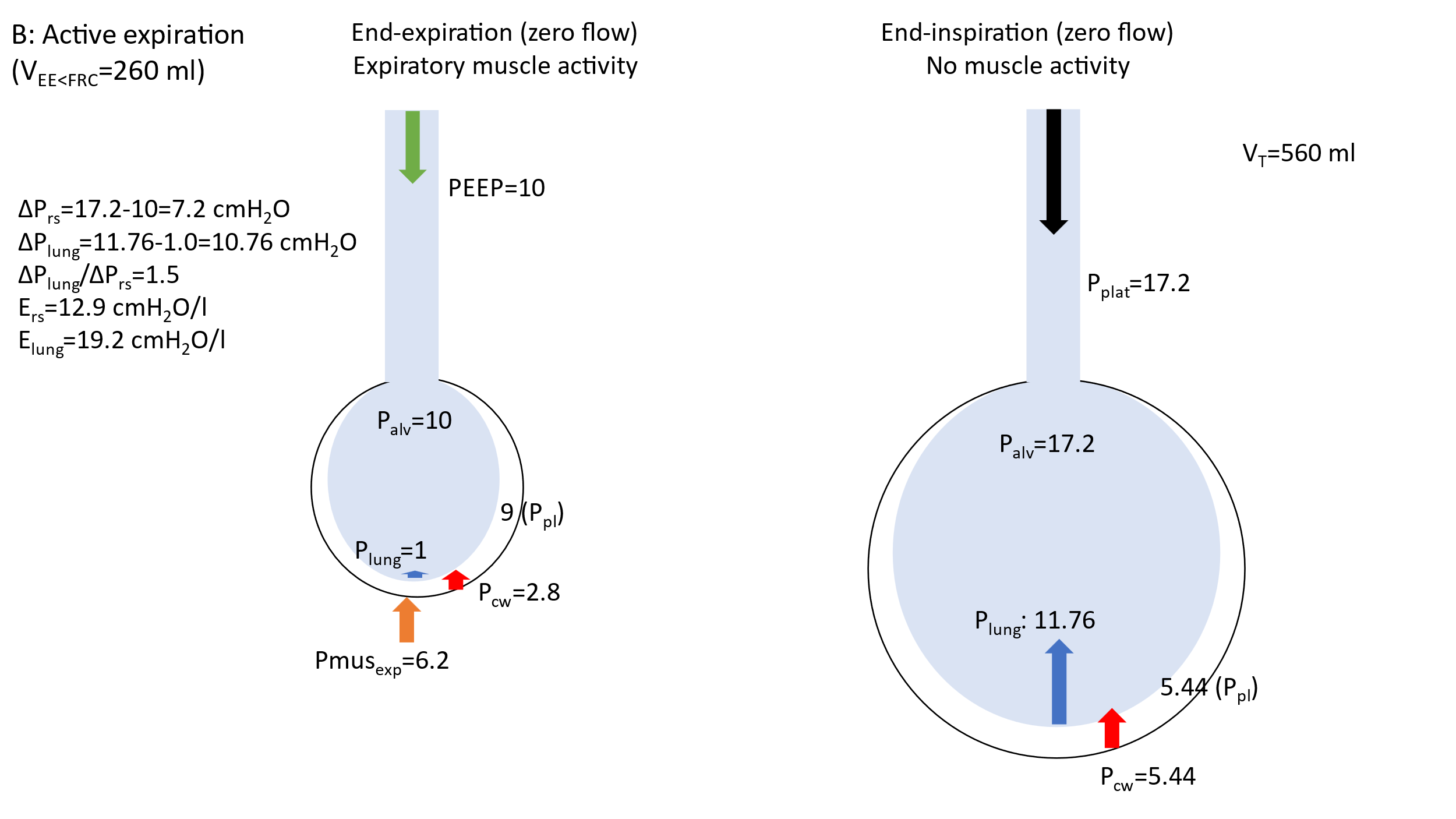


At end-expiration since flow is zero, PEEP (green arrow) and P_alv_ are equal. At this point set Pmus_exp_ is 6.2 cmH_2_O (orange arrow). The decrease in end-expiratory lung volume below that determined by PEEP (V_EE<FRC_) due to expiratory muscles activity, is set to 260 ml. At this volume P_cw_ is 2.8 cmH_2_O, 1.2 cmH_2_O lower (E_cw_xV_EE<FRC_=4.8x0.26=1.2) than that when expiration is passive (Fig. S1A). P_cw_=4-1.2=2.8 cmH_2_O (red arrow). P_pl_ is the algebraic sum of Pmus_EE_+P_cw_= 6.2+2.8=9 cmH_2_O. P_lung_=10-9=1 cmH_2_O (blue arrow). Notice that P_alv_ is the algebraic sum of P_cw_+Pmus_exp_+P_lung_=10 cmH_2_O. Assuming that the diaphragmatic contraction is able to increase volume above that determined by PEEP by 300 ml (since the beginning of diaphragmatic contraction is at volume below that determined by PEEP) V_T_ is 560 ml of which 300 is due to diaphragmatic activity and 260 ml is entered passively to the lung by relaxation of expiratory muscles^1^. Therefore, the end-inspiratory lung volume above that determined by PEEP is 300 ml. At this volume at absence of respiratory muscle pressure, P_plat_ equals to PEEP plus the increase in elastic recoil pressure of respiratory system due to 300 ml increase in volume above PEEP (P_plat_=PEEP+0.3x24=17.2 cmH_2_O, green arrow). ΔP_rs_=P_plat_-PEEP=7.2 cmH_2_O. The calculated E_rs_ decreases to 7.2/0.56=12.9 cmH_2_O/l and underestimates the actual E_rs_ (24 cmH_2_O/l) by 46%. P_cw_ is 5.44 cmH_2_O, 1.4 cmH_2_O higher that that at volume determined by PEEP (0.3x4.8=1.44). P_pl_ is 5.44 cmH2O, equals to P_cw_. P_lung_=P_alv_-P_pl_=17.2-5.44=11.76 cmH_2_O and ΔP_lung_=11.76-1=10.76 cmH_2_O. Notice that contrary to ΔP_rs_, the calculated E_lung_=[(11.76-1)/0.56=19.2 cmH_2_O] is similar to that in Fig. S1A. ΔP_lung_ takes into account the increase in P_lung_ due to passively increase in lung volume because of relaxation of expiratory muscles. ΔP_lung_/ΔP_res_=1.5, increased by 88% compared to absence of expiratory muscles activity during expiration.

^1^In this situation the V_T_ is largely unpredictable since at volumes below that determined by PEEP, the diaphragmatic contraction combined with expiratory muscles relaxation, decreases expiratory flow compared to that achieved by only relaxation of expiratory muscles. As a result, the volume determined by PEEP is reached faster and before complete relaxation of expiratory muscles, and this portion of diaphragmatic pressure (i.e. diaphragmatic pressure during V_EE_<FRC) is dissipated to decrease expiratory flow and not to increase inspiratory flow. Obviously, any value other than 300 ml can be used.
