# Supplementary material for: Driving pressure of respiratory system and lung stress in mechanically ventilated patients with active breathing
Source: Crit Care. 2024 Jan 12;28:19. doi: 10.1186/s13054-024-04797-3 (PMC10785492; doi:10.1186/s13054-024-04797-3)
Supplement: Supplementary file 2 — Additional file 2. Supplementary Methods, Results, Figures, Tables and References. [file 13054_2024_4797_MOESM2_ESM.docx]

**Additional file 2**

**Driving pressure of respiratory system and lung stress in mechanically ventilated patients with active breathing**

**Contents**

1. **Supplementary Methods**
2. Patients
3. Esophageal and gastric pressure insertion
4. Measurements/Calculations of respiratory variables
5. Details of the measurement of end-inspiratory elastic recoil pressure of respiratory system with PAV+
6. Estimation of expiratory muscle activity in patients with only esophageal catheters
7. Reasoning of choosing Elung to characterize deciles of 7-breath moving average measurements
8. **Supplementary Results**
9. **Supplementary Figures**
10. **Supplementary Tables**
11. **References**
12. **Supplementary Methods**
13. **Patients**

Out of the thirty-three patients initially enrolled in the study, two were excluded because their recording time was less than 1 hour; one patient developed distress almost immediately after switching to PAV+ and was placed on control mechanical ventilation, and in the second case, the primary physician reduced the assist level to less than 20% within 1 hour. Therefore, a total of 31 patients were included in the study. During the recording period, the treating physicians could change mode and settings at their best judgment. No written algorithms for ventilator settings were followed by the treating physicians. However, following the general principles of applying the level of assist and PEEP on PAV+ (1), is a common practice in our Intensive Care Unit.

1. **Esophageal and gastric pressure insertion**

The procedure for measuring esophageal and gastric pressure involved the employment of nasogastric catheter, endowed with esophageal and gastric balloons at the following steps (2):

1) The patient was positioned semi-recumbently. A local anaesthetic was applied in the nose.

2) Prior to insertion, the balloons were filled with air via three-way stopcock, until their complete inflation to verify their integrity.

3) The balloons were deflated and attached to a pressure transducer and a dedicated recording system.

4) The catheter was slowly advanced through the nose into the stomach, typically at a depth of 45 cm from the nostril. The procedure is comparable to inserting a nasogastric tube.

5) Following insertion, the esophageal balloon was filled with 4 or 0.5 ml of air for Nutrivent^TM^ and Cooper-Surgical catheters, respectively. During assisted spontaneous breathing, the esophageal pressure signal should exhibit negative swings concurrent with inspiratory muscle efforts. The esophageal pressure waveform was not smooth because of cardiac oscillations. The correct placement and appropriate filling of the esophageal balloon was verified with the Baydur test, as shown below. In summary, during an end-expiratory hold, the ratio of negative swings in airway (ΔPaw) and esophageal (ΔPes) pressures in response to inspiratory muscle contractions should be between 0.8-1.2. If not, the position of the esophageal balloon and/or the amount of air inside it should be corrected. Of note, the Baydur test was performed a) at least every 8 hrs. during the study, b) every time there was a change in patient position and c) whenever visual inspection of esophageal and gastric pressure waveforms suggested catheter movement or balloon deflation.


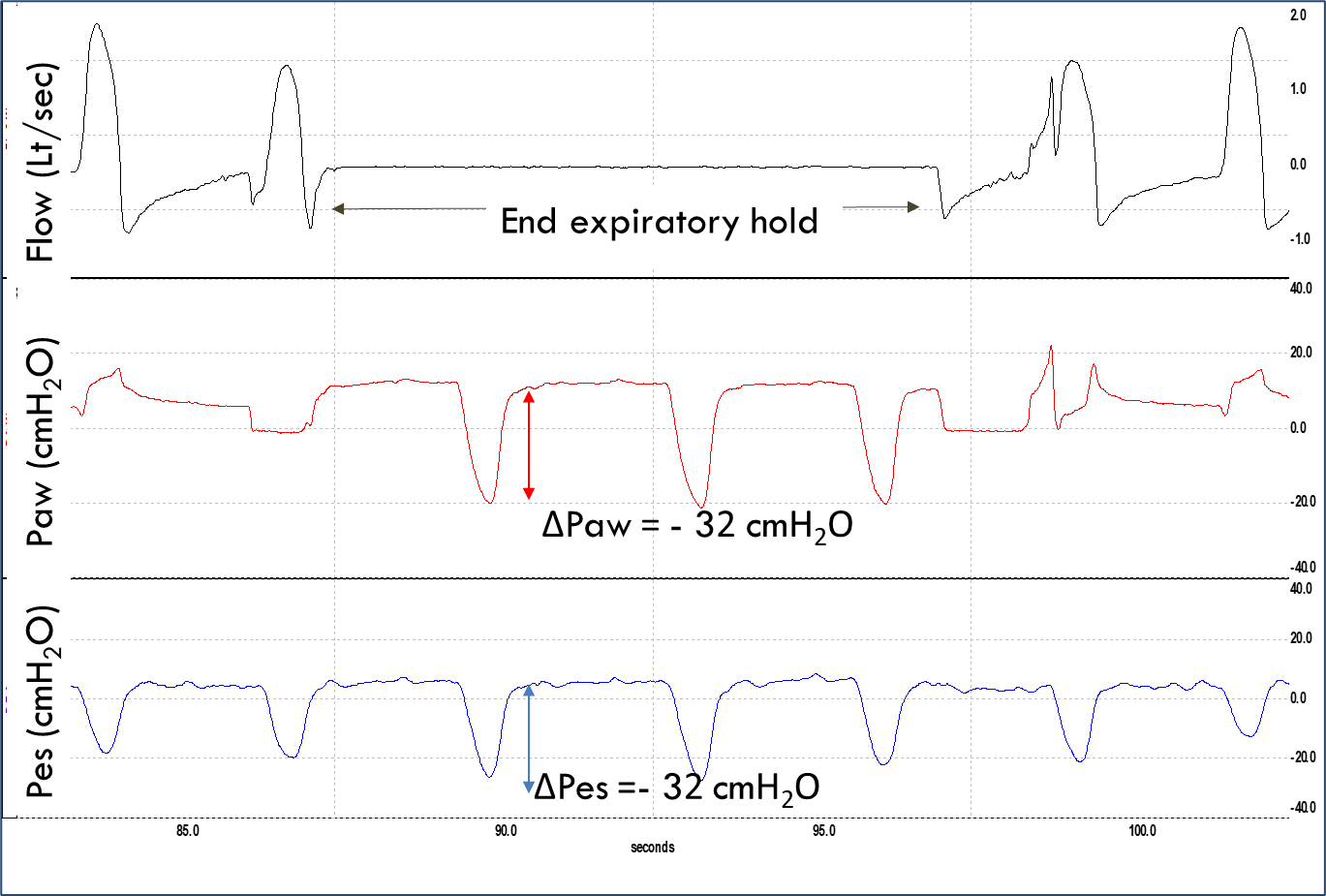


6) The next step was to fill the gastric balloon with the recommended amount of air, which was 4 ml for the Nutrivent^TM^ catheters that we used. Following gastric balloon inflation, the stomach was gently pressed, and a positive wave should be seen in the gastric pressure. The absence of cardiac oscillations and the positive deflection of gastric pressure during mechanical inflation implied intragastric balloon positioning. There is no calibration for gastric pressure monitoring and a final verification (usually not necessary) of intragastric balloon placement was performed by chest x-ray.

1. **Measurements/Calculations of Respiratory variables**

Airflow (V’), volume (V, time-integral of flow), airway pressure (Paw), esophageal pressure (Pes), gastric pressure (Pgas), dynamic transpulmonary pressure (P_lung_=Paw-Pes) and transdiaphragmatic pressure (Pdi=Pgas-Pes) were measured breath by breath. A heated pneumotachograph (Hans-Rudolf 3700, Kansas, USA) and differential pressure transducers (Micro-Switch 140PC, Honeywell, Ontario, Canada) were used to obtain V' and pressures, each signal sampled at 200 Hz (Windaq Instruments, Ohio, USA). Using a customized computer program, all the breaths in which 300 msc end-inspiratory occlusions applied, were identified and the end of expiration (zero flow, beginning of inspiration) and end of inspiration (zero flow, end of 300 msec occlusion) were marked. At these two points, Paw (Paw_EE_ and P_plat_) and Pes (Pes_EE_ and Pes_EI_) were measured and tidal volume (V_T_) of the occluded breath was calculated. Transpulmonary pressures at the end of expiration (P_LEE_) and inspiration (P_LEI_), driving pressure of total respiratory system (ΔP_rs_), driving transpulmonary pressure (ΔP_lung_), elastance of total respiratory system (E_rs_) and elastance of the lung (E_lung_) were measured as follows:

*ΔP_rs_= P_plat_-Paw_EE_*

*P_LEE_=Paw_EE_-Pes_EE_*

*P_LEI_=P_plat_-Pes_EI_*

*ΔP_lung_=P_LEI_-P_LEE_*

*Ers=ΔP_rs_/V_T_*

*E_lung_=ΔP_lung_/V_T_*

Pressure generated by all (inspiratory and expiratory) respiratory muscles (Pmus) was calculated from Pes considering the passive elastic and resistive properties of the chest wall. This calculation, which is based on the Campbell diagram, was described in detail earlier (3,4). Briefly, at each instant in the respiratory cycle Pmus is the difference between the Pes that would be obtained at the same volume and flow during passive inflation or deflation, and the Pes actually observed. With passive inflation or deflation, the Pes that would be obtained is given by:

*Pes(passive)=E_cw_xV+R_cw_xV’ [Eq: 1],*

where V is volume relative to the end-expiration, and E_cw_ and R_cw_ are, respectively, elastance and resistance of the chest wall. In all patients R_cw_ was assumed to be 1.5 cmH_2_O/l/sec (5,6), while E_cw_ was considered to be similar to that obtained during passive control mechanical ventilation, if the patient had such measurements, or if not, a value of 2.5% of predicted vital capacity was used. Inspiratory V' and expiratory V' were assigned positive and negative values, respectively. Thus, at time (t) from the beginning of mechanical inflation Pmus(t) was calculated as follows:

*Pmus(t)=E_cw_xV(t)+ R_cw_xV’(t) + Pes_EE_ -Pes(t), [Eq. 2]*

where V(t) is volume relative to that at end-expiration. Since during active breathing Pes at passive FRC was unknown, the contribution of inspiratory and expiratory muscle pressure is also unknown and for this reason the Pmus swings (peak-nadir) during the breath (ΔPmus_sw_) were reported.

The swings of Pdi (ΔPdi) during the inspiratory phase were measured as the difference between peak Pdi and Pdi at the point where Pdi started to increase rapidly (7). Dynamic transpulmonary pressure swings (Plung_sw_) during the breath were measured as the difference between the peak and nadir values. Mechanical inflation (T_I_) and deflation (T_E_) times were measured as the intervals between the beginning and the end of inspiratory and expiratory flow, respectively. Total breath duration (T_TOT_) was calculated as the sum of T_I_ and T_E_.

1. **Details of the measurement of end-inspiratory elastic recoil pressure of respiratory system with PAV+**

The ventilator software randomly applies a 0.3-sec end-inspiratory occlusion every 4-7 breaths and measures airway pressure at the end of occlusion (P_plat_). The scientific reasoning behind the validity of the measurement to correspond to the elastic recoil pressure of respiratory system at end-inspiration is described below (8):

With PAV+ at time t from the beginning of inspiration, inspiratory muscle pressure (Pmus_I_(t)) is given by the following equation:

*Pmus_I_(t)=V(t)x(Ers-%assistxErs/100)+V’(t)x(Rrs-%assistxRrs/100) [Eq. 3],*

where V(t) and V’(t) are inspiratory volume and flow at time t from the beginning of inspiration, respectively and Ers is elastance of respiratory system (1/Crs). During the rising phase of Pmus_I_, Pmus_I_ is always greater than the term V(t)x(Ers-%assist*Ers/100) and thus inspiratory flow is produced until peak Pmus_I_ is achieved (PeakPmus_I_). PeakPmus_I_ is given by:

*PeakPmus_I_=Vpeakx(Ers–%assistxErs/100)+V’peakx(Rrs-%assistxRrs/100) [Eq. 4],*

where Vpeak and V’peak are inspiratory volume and flow at peak Pmus, respectively. Thereafter Pmus_I_ declines and inspiratory flow continues, although in decreasing amounts. On the other hand, since there is inspiratory flow, the volume continues to rise, increasing the volume term V(t)x(Ers-%assistxErs). When Pmus_I_ is equal to the volume term there is no pressure available for flow and flow becomes expiratory flow driven by the elastic recoil pressure. In breaths selected for occlusion maneuver, an occlusion for 0.3 sec is applied when inspiratory flow reaches a value of 0.05 l/sec, (i.e., close to zero). The Pmus_I_ at the beginning of occlusion (zero flow) is:

*Pmus_I_=V_T_x(Ers-%assist*Ers/100) [Eq. 5],*

where V_T_ is tidal volume. Eq. 5 dictates that Pmus_I_ at the beginning of occlusion is low when V_T_ is low, and/or the % of assist is high. It has been shown that independent of respiratory drive, at the end of occlusion time (0.3 sec) Pmus_I_ has declined to baseline value, even if the rate of decline is very slow (i.e., 5 cmH2O/sec). At the end of occlusion expiratory muscle activity is usually absent or very low. Therefore, Paw at 0.3 sec of occlusion is similar to passive elastic recoil pressure corresponding to V_T_ of the occluded breath (8).

The small volume decrease (2-7 ml) during inspiratory valve closing time (≈50 msec) leads to a negligible underestimation of P_plat_ at the end of inspiration, and thus, it was disregarded.

1. **Estimation of expiratory muscles activity in patients with only esophageal catheters**

In patients for whom only Pes was measured, we identified the presence or absence of expiratory muscle activity during expiration through careful inspection of expiratory flow and Pes during this phase. Two signs which were unequivocally associated with expiratory muscle activity was carefully looking for (9). Firstly, an abrupt increase in expiratory flow, coupled with an increase in Pes, indicated expiratory muscle activity. Secondly, almost constant expiratory flow suggested a progressive increase in expiratory muscle pressure, countering the drop in elastic recoil due to volume decrease.

**6. Reasoning of choosing Elung to characterize deciles of 7-breath moving average (7-brMA) measurements**

Since the recording time varied among patients, we divided all the artifact-free 7-brMA measurements into deciles for each patient based on a progressive increase in E_lung_ (Decile 1 representing the lowest range of E_lung_, and Decile 10 representing the highest range of E_lung_). Consequently, each patient was characterized by 10 segments, each containing an equal number of 7-brMA measurements, with progressively increasing E_lung_ values that did not overlap. E_lung_ was chosen to characterize each decile because change in this variable is associated with corresponding change in ventilatory demands (10). Furthermore, E_lung_ is a key determinant of ΔPlung, reflecting the degree of lung stretch at a given volume, sensed by the mechanoreceptors of the control of breathing system (11-12).

1. **Supplementary Results**

Two out of thirty-one patients were placed on t-piece within the first 24 hours after switching to PAV+. In these patients the recording time on PAV+ was 18.1 and 7.3 hours, respectively. One patient failed on PAV+ after 12.5 hours and was switched to control mode. In two patients the recording was interrupted for procedural reasons (recording time 10.7 and 7.3 hours, respectively). Recording data during the routine day care (i.e., washing/cleaning/skin-mouth care) was not analyzed.

Since PEEPi in this patient population was negligible (<0.5 cmH_2_O), we did not correct ΔP_rs_ for it. Similarly, we did not correct for the small increase in ΔPgas (<1.5 cmH_2_O), observed occasionally during occluded breaths in some patients. The uncorrected ΔPrs for PEEPi and ΔPgas are higher than the corrected values. Consequently, using uncorrected ΔPrs leads to a slight overestimation of ΔPlung, although the effect is minimal.

**III. Supplementary Figures**

**Figure S2**


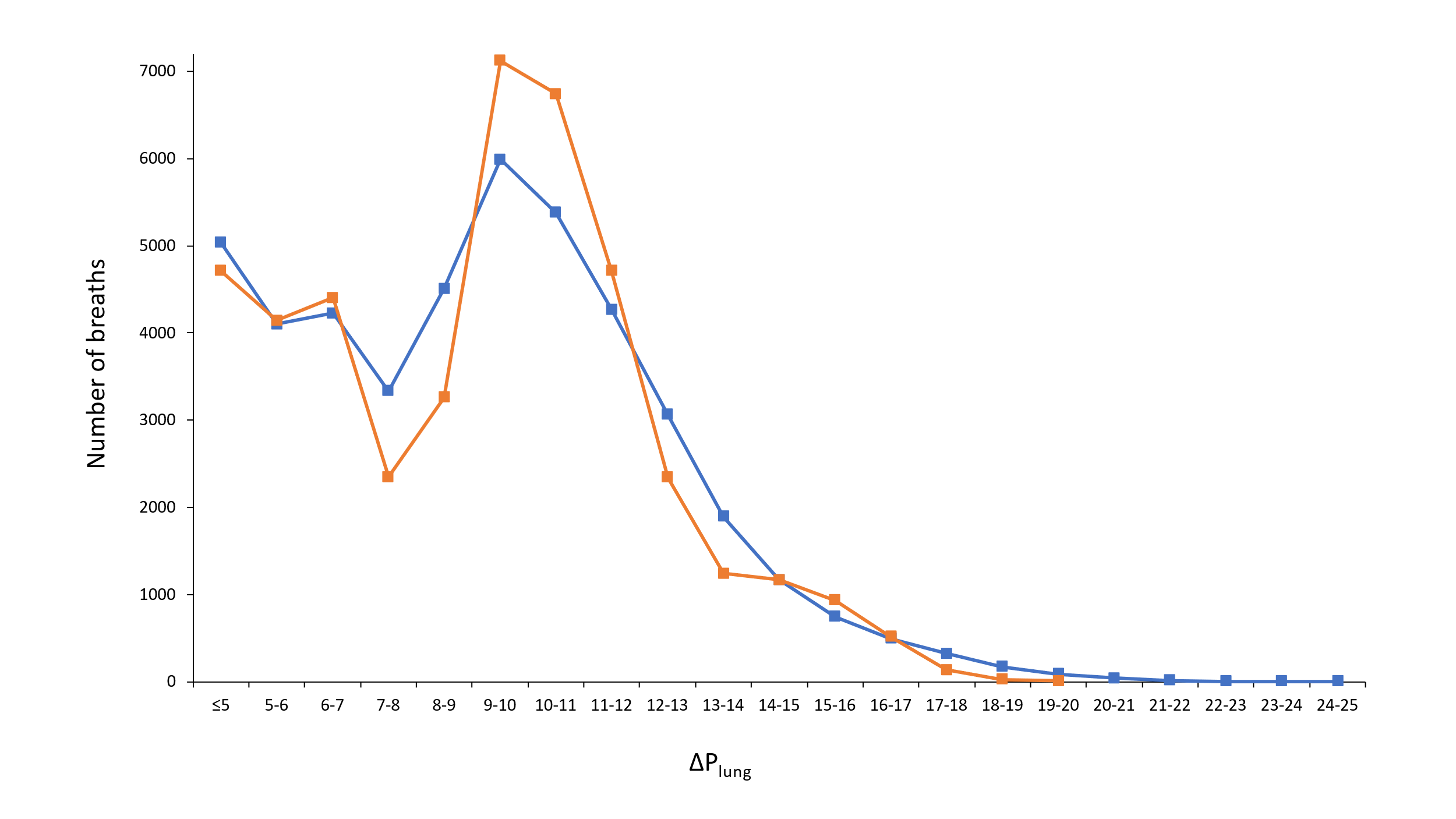


Figure S2: Number of occluded breaths (blue) and of 7-breath moving average measurements (orange) with ΔP_lung_ within the range of each cmH_2_O from less than 5 cmH_2_O to maximum value.

**Figure S3**


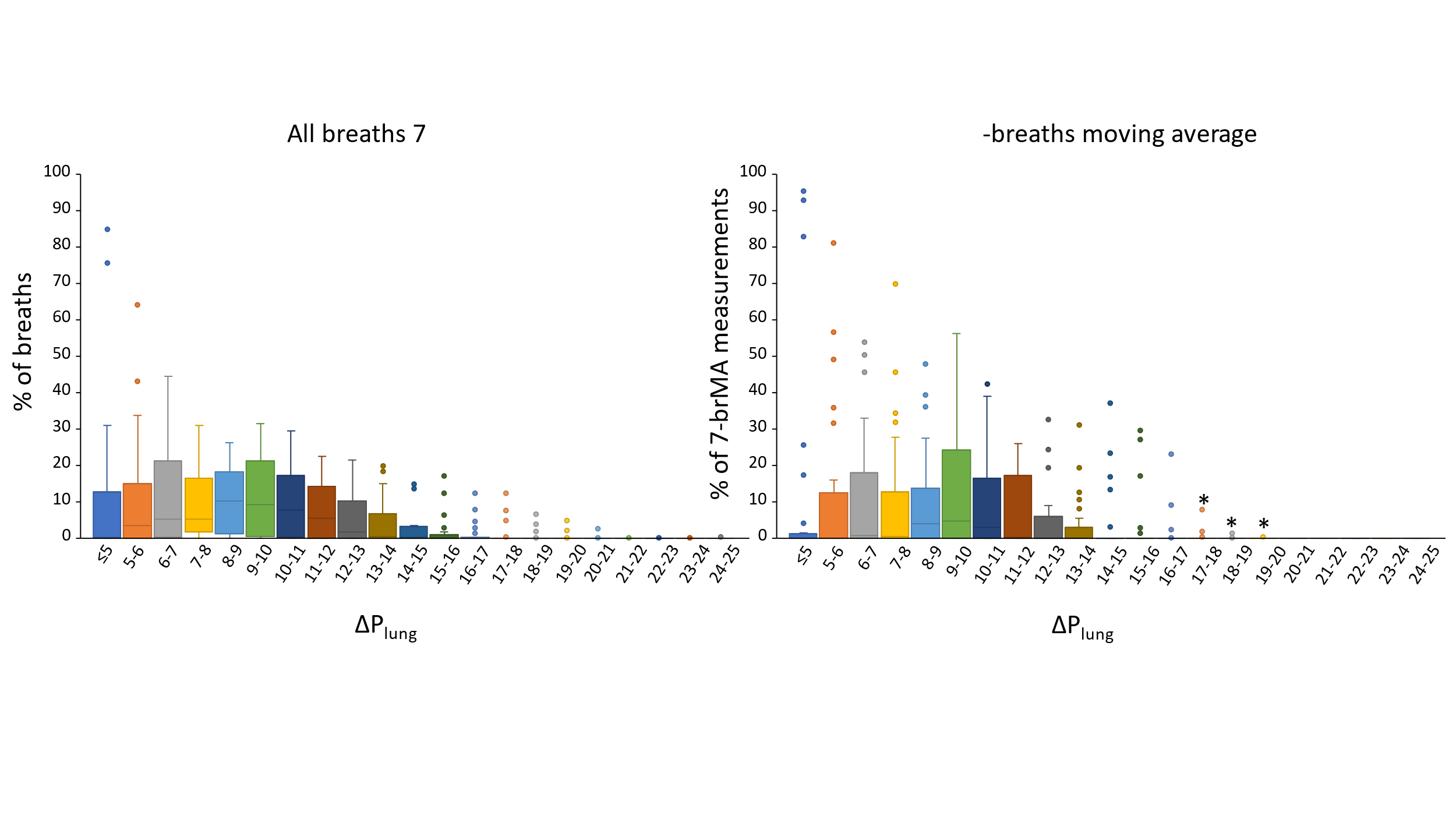


Figure S3: Box and whisker plots of number of occluded breaths (left) and of 7-breaths moving average measurements (right), expressed as % of total, with ΔP_lung_ within the range of each cmH_2_O from less than 5 cmH_2_O to maximum values. Outliers are shown by circles. Each color represents a specific pressure range

*Significantly different from % of total number of occluded breaths at a given ΔP_lung_ range (Wilcoxon paired test).

**Figure S4**


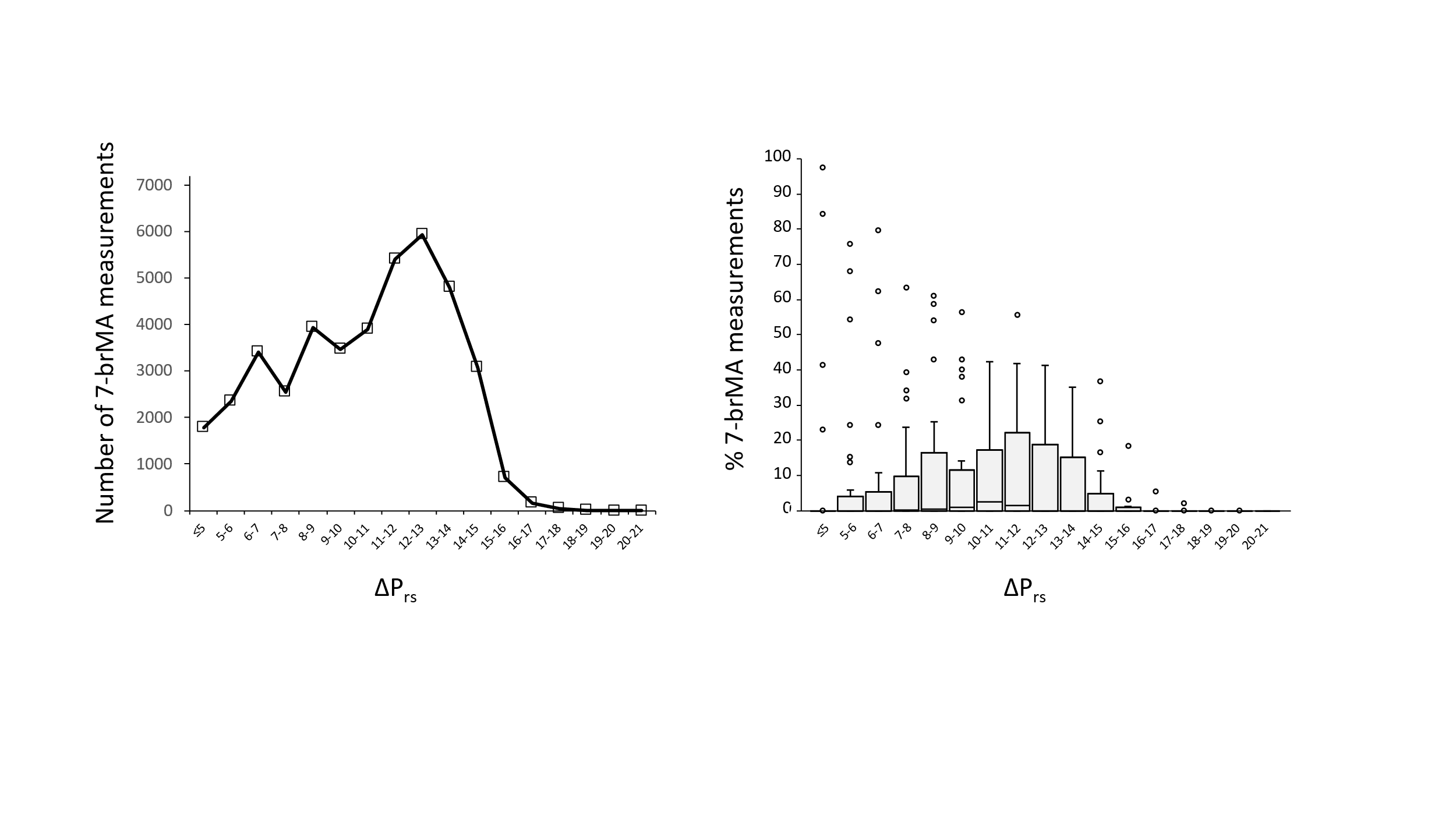


Figure S4: Number of 7-breath moving average measurements (A) and % of total measurements (B) with ΔP_rs_ within the range of each cmH_2_O from ≤5 cmH_2_O to maximum values. Outliers are shown by circles.

**Figure S5**

**
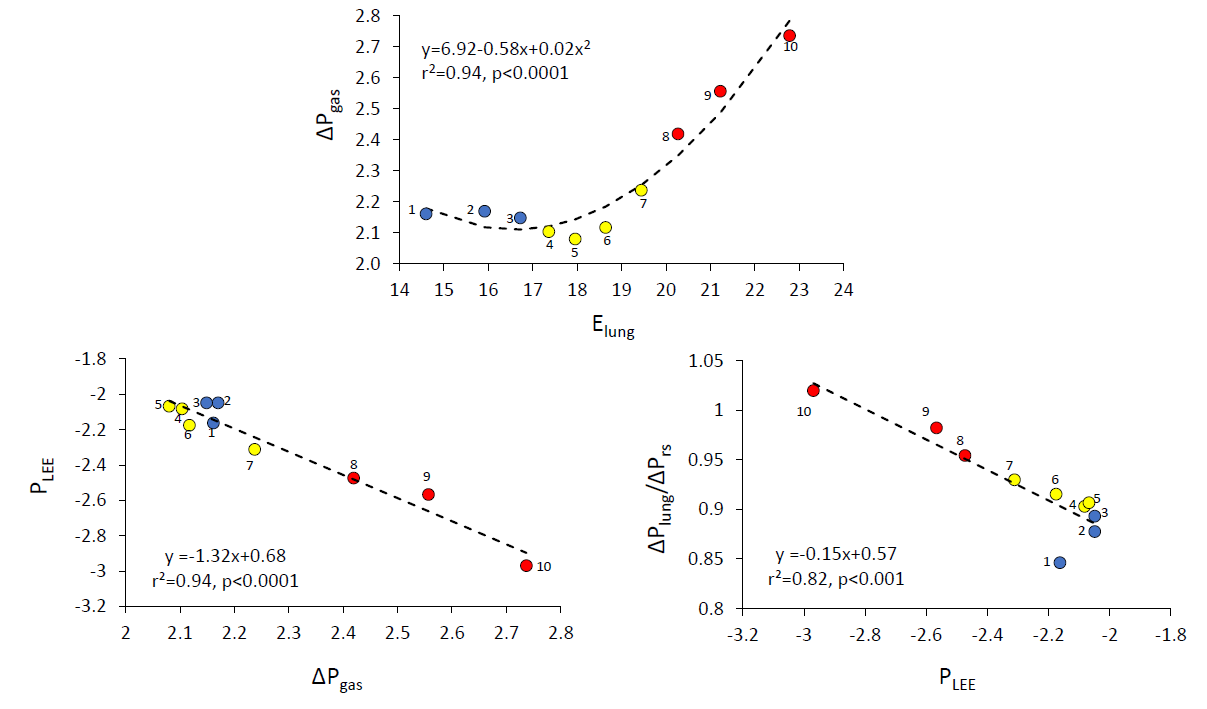
**

Figure S5: Relationship between 1) the increase in lung elastance (E_lung_) and gastric pressure increase during expiration (ΔP_gas_) (Α), 2) ΔP_gas_ and transpulmonary pressure at the end of expiration (P_LEE_) (Β) and 3) P_LEE_ and ΔP_lung_/ΔP_rs_ in 22 patients instrumented with esophageal and gastric catheters. Each circle represents the average values of these variables in each of the 10 segments characterized by increasing E_lung_. Blue circles: Deciles 1-3 (low E_lung_). Yellow circles: Deciles 4-7 (moderate E_lung_). Red circles: Deciles 8-10 (high E_lung_). Notice that high E_lung_ is associated with the larger increase in ΔP_gas_ and the lowest P_LEE_, causing the highest increase in ΔP_lung_/ΔP_rs_. Observe also that ΔP_gas_ remains rather constant from decile one to six, increasing considerably after that. This is reflected in almost constant P_LEE_ and ΔP_lung_/ΔP_rs_ from decile one to six. Numbers beside each circle denote the decile.

**Figure S6**


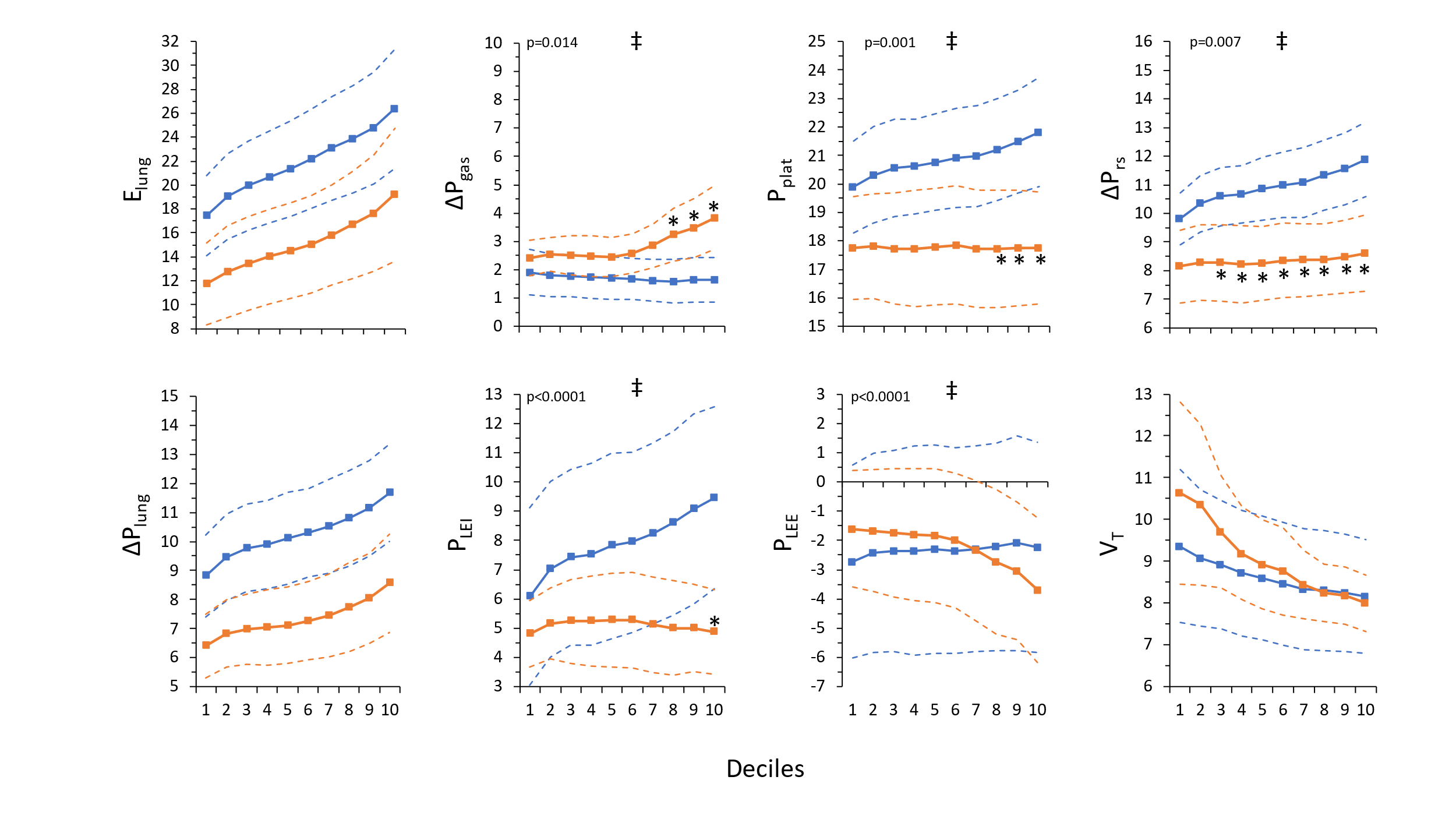


Figure S6: Patients instrumented with both esophageal and gastric catheters (n=22). Effects of a progressive increase in lung elastance (E_lung_, Decile 1: the lowest E_lung_; Decile 10 the highest E_lung_) on average respiratory variables in Group A (blue squares connected by blue lines, n=11) and Group B (orange squares connected by orange lines, n=11). Blue and orange dashed lines indicate SD range in Group A and B, respectively. Notice the significant interaction between groups in transpulmonary pressure at end-inspiration (P_LEI_) and end-expiration (P_LEE_), driving pressure (P_rs_), end-inspiratory plateau pressure (P_plat_) and gastric pressure increase during expiration (ΔP_gas_).

ⱡSignificant interaction between Groups (Split-plot ANOVA).

*Significant difference from the corresponding value of Group A (comparisons were performed if interaction between groups was significant).

**IV. Supplementary Tables**

**Table S1: Details on the day of recording**

| Duration of recording, hrs. | 16.9 (7.3-22.5) |  |
| --- | --- | --- |
| Analyzed number of occluded breaths | 970 (599-2257) |  |
| PAV+ assist, % (start-end) | 50 (40-64) – 45 (40-60) | p=0.224 |
| PEEP, cmH_2_O (start-end) | 9 (8-10) – 9 (8-10) | p=0.518 |
| PaO_2_/FIO_2_ | 231 (211-255) |  |
| PaO_2_, mmHg | 88 (80-92) |  |
| PaCO_2_, mmHg | 44 (38-48) |  |
| pH | 7.42 (7.39-7.44) |  |
| SOFA score, at the day of recording | 6 (4-8) |  |
| Propofol, , mcg/kg/min, [n=11] | 17.5 (16.4-18.0) |  |
| Dexmedetomidine, mcg/kg/min, [n=18] | 0.40 (0.0-0.58) |  |
| Midazolam, mcg/kg/min mg/hr, [n=3] | 0.7 (0.7-0.9) |  |
| Remifentanil, mcg/kg/min, [n=21] | 0.05 (0.04-0.07) |  |
| Fentanyl, mcg/kg/min, [n=4] | 0.02 (0.02-0.02) |  |
| Noradrenaline, mcg/kg/min, [n=18] | 0.04 (0.03-0.07) |  |
| Switch to t-piece*, Yes/No | 2/29 |  |
| Switch to CMV*, Yes/No | 2/29 |  |
| Procedural recording interruption | 2/29 |  |

Values are median and interquartile range (IQR) or counts. SOFA; Sequential Organ Failure Assessment. CMV; Controlled Mechanical Ventilation.

Number in bracket indicate the number of patients receiving the specific medication.

*During the recording time.

|  | Median value (IQR) | Median CV (IQR) |
| --- | --- | --- |
| P_plat_, cmH_2_O | 19.4 (17.0-22.1) | 4.4 (3.5-5.8) |
| PEEP, cmH_2_O | 9.6 (8.1-10.3) | 3.1 (2.0-5.8) |
| P_LEI_, cmH_2_O | 6.3 (3.8-7.9) | 21.0 (14.6-36.2) |
| P_LEE_, cmH_2_O | -2.24 (-4.8—0.1) | 38.9 (20.3-78.2) |
| ΔP_rs_, cmH_2_O | 9.7 (7.2-12.0) | 8.6 (7.0-11.5) |
| ΔP_lung_, cmH_2_O | 8.8 (6.4-10.5) | 10.7 (8.5-14.5) |
| V_T_, ml/kg | 9.1 (7.7-11.4) | 10.1 (6.6-13.1) |
| E_rs_, cmH_2_O/l | 18.7 (12.1-22.6) | 15.3 (9.1-19.8) |
| E_lung_, cmH_2_O/l | 16.5 (11.4-21.9) | 13.5 (10.4-17.1) |
| ΔP_lung_/ΔP_rs_ | 0.88 (0.82-1.04) | 8.6 (7.3-14.0) |
| T_TOT_, sec. | 3.02 (2.61-3.59) | 9.4 (7.0-14.4) |
| T_I_, sec. | 1.11 (1.04-1.20) | 7.9 (6.1-10.2) |
| T_I_/T_TOT_ | 0.41 (0.36-0.42) | 5.9 (4.6-6.5) |
| Plung_sw_, cmH_2_O | 14.8 (12.5-16.4) | 9.2 (7.7-12.6) |
| Pmus_sw_, cmH_2_O | 13.0 (11.7-16.6) | 10.9 (8.5-15.1) |
| ΔP_gas_, cmH_2_O | 2.10 (0.69-3.87) | 28.6 (16.7-42.6) |
| ΔPdi, cmH_2_O | 8.02 (7.3-9.6) | 18.0 (14.5-25.4) |

**Table S2: Median values and coefficient of variations (interquartile range) of 7-breaths moving average measurements of various variables during the whole recording period.**

Values are median and interquartile range (IQR). P_plat_; End-inspiratory plateau pressure. PEEP; Positive end-expiratory pressure. P_LEI_, P_LEE_; Transpulmonary pressure at the end of inspiration and expiration, respectively. ΔP_rs_, ΔP_lung_; Driving pressure of respiratory system and lung, respectively. V_T_; Tidal volume. E_rs_, E_lung_; Elastance of respiratory system and lung, respectively. T_TOT_; Total breath duration. T_I_; Mechanical inflation time. Plung_sw_, Pmus_sw_; Dynamic and respiratory muscles pressure swings, respectively. ΔP_gas_; Rise in P_gas_ during expiration. ΔPdi; Transdiaphragmatic pressure swings during inspiration. CV; coefficient of variation.

**Table S3: Respiratory variables with increasing lung elastance (Elung)**

| **Decile**  **E_lung_**  **cmH_2_O/L** | **1**  **12.9**  **(9.1-18.2)** | **2**  **13.7**  **(10.0-19.6)** | **3**  **14.3**  **(10.7-20.4)** | **4**  **14.7**  **(11.4-21.0)** | **5**  **15.1**  **(11.8-21.8)** | **6**  **15.4**  **(12.1-22.8)** | **7**  **17.2**  **(12.3-23.3)** | **8**  **17.6**  **(12.6-24.1)** | **9**  **18.1**  **(13.0-25.5)** | **10**  **19.5**  **(13.8-26.8)** | **p** |
| --- | --- | --- | --- | --- | --- | --- | --- | --- | --- | --- | --- |
| Pplat  cmH_2_O | 18.5  (16.7-21.4) | 19.3  (16.7-21.5) | 19.4  (16.8-21.7) | 19.6  (17.0-21.8) | 19.5  (17.0-22.3) | 19.5  (17.1-22.4) | 19.4  (16.7-22.7) | 19.9  (16.8-22.6) | 20.0  (16.9-22.8) | 19.9  (17.2-22.9) | <0.001 |
| PEEP  cmH_2_O | 9.7  (8.0-10.7) | 9.8  (8,1-10.5) | 9.7  (8.1-10.4) | 10.0  (8.1-10.4) | 9.9  (8.0-10.3) | 9.7  (7.9-10.2) | 9.6  (8.0-10.40 | 9.6  (8.0-10.3) | 9.5  (7.9-10.3) | 9.6  (7.9-10.2) | >0.05 |
| V_T_  ml/kg | 9.2  (7.8-13.0) | 9.2  (7.6-12.3) | 9.1  (7.6-11.9) | 8.9  (7.1-11.4) | 8.7  (7.1-11.3) | 8.6  (7.1-11.0) | 8.4  (6.8-10.6) | 8.4  (6.9-9.8) | 8.4  (7.0-9.5) | 8.2  (6.8-9.4) | 0.002 |
| T_TOT_  sec | 3.26  (2.67-4.0) | 3.21  (2.65-3.78) | 3.10  (2.64-3.78) | 3.04  (2.63-3.71) | 2.94  (2.55-3.56) | 2.91  (2.55-3.48) | 2.87  (2.45-3.47) | 2.90  (2.39-3.37) | 2.92  (2.40-3.50) | 2.85  (2.31-3.49) | 0.007 |
| T_I_  sec | 1.20  (1.03-1.28) | 1.18  (1.05-1.27) | 1.15  (1.05-1.22) | 1.13  (1.05-1.21) | 1.10  (1.05-1.21) | 1.07  (1.02-1.18) | 1.07  (1.00-1.20) | 1.08  (1.00-1.18) | 1.09  (0.98-1.15) | 1.04  (0.97-1.13) | 0.000 |
| Plung_sw_  cmH_2_O | 12.4  (9.9-15.0) | 13.5  (9.9-15.7) | 13.2  (10.0-15.8) | 13.8  (10.0-16.0) | 13.8  (10.1-16.1) | 13.9  (10.1-16.3) | 13.9  (10.2-16.6) | 14.2  (11.2-16.9) | 15.0  (11.4-17.4) | 15.2  (12.4-18.4) | <0.001 |
| Pmus_sw_  cmH_2_O | 11.9  (9.9-15.4) | 12.3  (10.8-16.1) | 12.0  (11.0-15.2) | 12.1  (11.1-15.2) | 12.7  (10.9-15.1) | 12.8  (10.7-15.6) | 12.9  (10.9-15.7) | 13.1  (11.0-16.7) | 13.1  (11.2-18.3) | 14.0  (11.5-19.0) | 0.001 |

**Table S3; cont. Values are median and interquartile range (IQR). See Table S2 for definition of abbreviations.**

| ΔPgas  cmH_2_O | 2.06  (1.25-3.02) | 2.14  (1.06-3.02) | 1.97  (0.99-3.30) | 1.92  (0.80-3.39) | 2.05  (0.74-3.27) | 2.13  (0.77-3.30) | 2.18  (0.65-3.71) | 2.26  (0.44-4.13) | 2.51  (0.40-2.39) | 2.55  (0.53-4.77) | >0.05 |
| --- | --- | --- | --- | --- | --- | --- | --- | --- | --- | --- | --- |
| ΔPdi  cmH_2_O | 7.49  (6.00-8.53) | 7.70  (6.64-8.95) | 7.95  (6.99-9.13) | 7.92  (7.18-9.15) | 8.00  (7.31-9.13) | 8.25  (7.21-9.37) | 7.97  (7.14-9.70) | 8.11  (6.68-9.39) | 8.24  (8.23-9.40) | 9.13  (6.59-10.64) | 0.028 |
| PLEI  cmH_2_O | 5.88  (2.95-7.32) | 6.03  (3.26-7.80) | 6.47  (3.52-8.17) | 6.51  (3.47-8.16) | 6.66  (3.53-8.37) | 6.57  (3.77-8.81) | 6.66  (3.71-8.39) | 6.94  (3.87-8.66) | 7.17  (3.92-8.78) | 7.05  (4.69-8.75) | 0.01 |
| PLEE  cmH_2_O | -2.15  (-4.48-0.77) | -2.18  (-4.61-0.82) | -1.83  (-4.68-0.93) | -1.62  (-4.71-1.03) | -1.48  (-4.43-0.92) | -1.63  (-5.24-0.79) | -2.10  (-5.06-0.42) | -2.67  (-5.25-0.68) | -2.81  (-5.50-0.82) | -3.14  (-6.43—0.05) | 0.027 |
| ΔP_rs_  cmH_2_O | 9.38  (7.09-11.20) | 9.57  (7.01-11.43) | 9.59  (7.05-11.63) | 9.44  (6.60-11.54) | 9.59  (6.56-12.15) | 9.55  (6.78-12.44) | 9.48  (6.85-12.47) | 9.90  (7.18-12.09) | 9.95  (7.47-12.02) | 10.27  (7.77-12.83) | <0.001 |
| ΔP_lung_  cmH_2_O | 7.97  (5.67-9.95) | 8.45  (5.89-10.53) | 8.57  (5.98-10.15) | 8.47  (5.83-10.39) | 8.63  (5.94-10.33) | 8.81  (6.05-10.59) | 9.20  (6.31-10.81) | 9.51  (6.66-11.32) | 9.78  (6.85-11.94) | 10.14  (7.43-12.95) | <0.001 |
| ΔP_lung_/ΔP_rs_ | 0.87  (0.73-0.99) | 0.88  (0.74-1.01) | 0.91  (0.74-1.03) | 0.90  (0.76-1.04) | 0.88  (0.77-1.01) | 0.89  (0.77-1.03) | 0.90  (0.81-1.05) | 0.90  (0.82-1.07) | 0.92  (0.83-1.10) | 0.96  (0.83-1.13) | 0.002 |
| E_rs_  cmH_2_O/L | 16.1  (9.9-21.8) | 16.8  (9.9-21.8) | 17.4  (10.3-22.2) | 17.8  (11.0-22.8) | 18.24  (12.2-22.8) | 19.0  (12.6-23.3) | 20.1  (13.1-23.6) | 20.2  (13.5-24.2) | 20.3  (14.0-25.5) | 20.7  (14.8-27.3) | <0.001 |

**Table S4: Respiratory variables at progressively increasing lung elastance in group A (n=17) and group B (n=14)**

| **Decile** | | **1** | **2** | **3** | **4** | **5** | **6** | **7** | **8** | **9** | **10** | **p*** |
| --- | --- | --- | --- | --- | --- | --- | --- | --- | --- | --- | --- | --- |
| PEEP,  cmH_2_O | **Group A**  **Group B** | 9.2 (8.6-10.7)  9.9 (7.3-10.5) | 9.8 (8.4-10.6)  9.8 (7.3-10.3) | 9.7 (8.3-10.6)  9.8 (7.4-20.3) | 10.0 (8.3-10.6)  9.8 (7.4-10.3) | 9.9 (8.2-10.5)  9.7 (7.6-10.3) | 10.0 (8.1-10.5)  9.6 (7.5-10.2) | 10.0 (8.1-10.5)  9.6 (7.5-10.3) | 9.9 (8.1-10.5)  9.6 (7.4-10.3) | 9.9 (8.1-10.4)  9.5 (7.4-10.3) | 9.9 (8.1-10.4)  9.6 (7.2-10.2) | 0.364 |
| ΔPdi,  cmH_2_O | **Group A**  **Group B** | 7.4 (6.1-8.9)  7.6 (4.8-8.4) | 7.9 (6.7-9.0)  7.6 (6.5-8.3) | 8.2 (7.0-9.2)  7.7 (7.0-9.1) | 7.9 (7.1-9.0)  8.0 (7.2-9.5) | 8.0 (8.0-9.1)  8.0 (7.1-9.4) | 8.3 (7.6-9.5)  7.9 (6.8-9.3) | 8.3 (7.8-11.8)  7.8 (6.9-9.1) | 8.3 (7.5-11.8)  7.1 (6.4-9.0) | 8.6 (7.5-11.6)  7.4 (5.0-8.9) | 9.5 (8.2-11.7)  8.8 (4.9-9.3) | 0.271 |
| Plung_sw_,  cmH_2_O | **Group A**  **Group B** | 13.8 (10.0-16.5)  12.3 (9.5-14.4) | 14.1 (10.6-17.3)  13.2 (9.6-14.5) | 14.6 (11.0-17.4)  13.1 (9.1-114.9) | 14.5 (11.2-17.6)  13.5 (8.7-15.3) | 14.7 (11.4-18.3)  13.5 (8.9-15.4) | 15.1 (11.5-18.4)  13.9 (9.0-15.7) | 15.0 (11.4-18.5)  13.6 (9.1-15.8) | 15.4 (11.6-18.8)  14.0 (9.2-16.2) | 15.5 (11.7-19.0)  14.6 (10.3-16.5) | 15.9 (12.7-20.1)  15.1 (10.3-17.9) | 0.849 |
| Pmussw,  cmH_2_O | **Group A**  **Group B** | 10.7 (9.1-15.4)  11.7 (10.6-14.7) | 11.3 (9.2-15.8)  12.2 (11.1-15.4) | 11.6 (9.4-16.1)  12.7 (11.2-14.9) | 11.6 (9.4-16.7)  12.5 (11.6-14.9) | 11.8 (9.4-17.0)  12.5 (11.9-14.9) | 11.9 (9.5-17.6)  13.1 (11.2-15.5) | 12.0 (9.5-17.7)  13.1 (11.3-15.4) | 12.2 (9.6-19.4)  13.3 (11.4-16.3) | 12.8 (9.7-19.7)  13.6 (11.4-17.6) | 13.3 (10.1-20.2)  14.6 (12.4-18.3) | 0.764 |
| T_TOT_,  sec | **Group A**  **Group B** | 3.26 (2.70-3.35)  3.28 (2.58-3.50) | 3.24 (2.70-3.96)  3.23 (2.57-3.76) | 3.05 (2.70-3.89)  3.19 (2.55-3.69) | 3.02 (2.67-3.86)  3.12 (2.54-3.75) | 2.95 (2.56-3.57)  2.99 (2.49-3.65) | 2.97 (2.57-3.57)  2.97 (2.43-3.49) | 2.92 (2.50-3.60)  2.93 (2.44-3.47) | 2.96 (2.49-3.49)  2.95 (2.38-3.49) | 2.92 (2.46-3.50)  2.95 (2.33-3.53) | 2.86 (2.34-3.50)  2.83 (2.25-3.43) | 0.607 |
| T_I_,  sec | **Group A**  **Group B** | 1.20 (1.00-1.36)  1.21 (1.06-1.25) | 1.20 (1.01-1.56)  1.18 (1.07-1.22) | 1.18 (1.00-1.36)  1.15 (1.09-1.20) | 1.17 (1.00-1.32)  1.13 (1.08-1.18) | 1.12 (0.99-1.28)  1.10 (1.05-1.16) | 1.13 (0.99-1.26)  1.07 (1.04-1.16) | 1.14 (0.98-1.26)  1.07 (1.02-1.14) | 1.14 (0.97-1.24)  1.06 (1.00-1.14) | 1.12 (0.95-1.29)  1.06 (0.99-1.12) | 1.06 (0.93-1.33)  1.03 (0.97-1.12) | 0.291 |

Values are median and interquartile range (IQR). See Table S2 for definition of abbreviations.

*p value for interaction between groups (Split-plot ANOVA).

**Table S5. Ventilatory variables during periods with ΔPlung<12 and ≥12 cmH_2_O in 14 patients exhibited both such periods**

|  | ΔPlung<12 cmH_2_O | ΔPlung≥12 cmH_2_O | p |
| --- | --- | --- | --- |
| % of 7-bMA measurements | 86.38 (30.9-95.4) | 13.63 (4.6-69.1) | 0.035 |
| Pplat, cmH_2_O | 21.00 (17.83-22.57) | 22.34 (19.96-24.39) | 0.002 |
| PEEP, cmH_2_O | 10.08 (8.24-11.55) | 10.01 (8.24-11.92) | NS |
| P_LEI_, cmH_2_O | 7.08 (1.27-8.46) | 7.71 (4.00-11.21) | 0.003 |
| P_LEE_, cmH_2_O | -3.18 (-8.40—1.56) | -4.95 (-9.96—2.57) | 0.026 |
| ΔP_rs_, cmH_2_O | 10.89 (9.39-12.27) | 12.60 (11.31-13.98) | 0.002 |
| ΔP_lung_, cmH_2_O | 10.39 (9.55-11.05) | 12.68 (12.38-) | 0.001 |
| ΔP_lung_/ΔP_rs_ | 0.96 (0.81-1.10) | 1.07 (0.93-1.25) | 0.002 |
| E_rs_, cmH_2_O/l | 20.02 (16.98-25.37) | 23.28 (16.54-29.46) | NS |
| E_lung_, cmH_2_O/l | 20.96 (15.87-25.20) | 25.11 (18.94-30.63) | 0.001 |
| V_T_, ml/kg | 8.33 (6.80-12.11) | 8.97 (7.32-12.18) | 0.03 |
| T_TOT_, sec | 2.93 (2.56-3.46) | 2.87 (2.41-3.38) | NS |
| T_I_, sec | 1.15 (1.01-1.41) | 1.13 (0.93-1.39) | NS |
| T_I_/T_TOT_ | 0.39 (0.37-0.42) | 0.39 (0.37-0.41) | NS |
| Plung_sw_, cmH2O | 14.81 (12.93-17.11) | 17.70 (15.81-20.73) | 0.001 |
| Pmus_sw_, cmH2O | 12.36 (9.97-17.66) | 13.31 (11.78-21.35) | 0.002 |
| ΔPdi, cmH2O | 7.88 (5.67-8.15) | 8.29 (6.38-10.53) | 0.005 |
| ΔPgas, cmH2O | 2.93 (0.73-3.99) | 3.82 (0.66-4.73) | NS |
| ΔPdi+ΔPgas, cmH2O | 10.05 (8.56-11.8) | 11.25 (9.66-13.44) | 0.009 |

Values are median and interquartile range (IQR). See Table S2 for definition of abbreviations.

**V. References**

1. Xirouchaki N, Kondili E, Vaporidi K, Xirouchakis G, Klimathianaki M, Gavriilidis G, et al. Proportional assist ventilation with load-adjustable gain factors in critically ill patients: Comparison with pressure support. Intensive Care Med. 2008;34(11):2026–34.

2. Akoumianaki E, Maggiore SM, Valenza F, Bellani G, Jubran A, Loring SH, et al. The application of esophageal pressure measurement in patients with respiratory failure. Am J Resp Crit Care Med. 2014;189:520–31.

3. Georgopoulos D, Mitrouska I, Bshouty Z, Webster K, Patakas D, Younes M. Respiratory Response to CO 2 during Pressure-support Ventilation in Conscious Normal Humans. Am J Respir Crit Care Med. 1997;157(1):146-54.

4. Ceorgopoulos D, Mitrouska I, Webster K, Bshouty Z, Younes M. Effects of inspiratory muscle unloading on the response of respiratory motor output to CO2. Am J Respir Crit Care Med. 1997;155(6):2000–9.

5. Xirouhaki N, Kondili E, Mitrouska I, Siafakas N, Georgopoulos D. [Response of respiratory motor output to varying pressure in mechanically ventilated patients.](https://pubmed-ncbi-nlm-nih-gov.uml.idm.oclc.org/10543268/) Eur Respir J. 1999; 14(3):508-16.

5. Kondili E, [Prinianakis](https://pubmed-ncbi-nlm-nih-gov.uml.idm.oclc.org/?term=Prinianakis+G&cauthor_id=11534562) G, [Anastasaki](https://pubmed-ncbi-nlm-nih-gov.uml.idm.oclc.org/?term=Anastasaki+M&cauthor_id=11534562) M, [Georgopoulos](https://pubmed-ncbi-nlm-nih-gov.uml.idm.oclc.org/?term=Georgopoulos+D&cauthor_id=11534562) D. Acute effects of ventilator settings on respiratory motor output in patients with acute lung injury. Intensive Care Med. 2001;27(7):1147-57.

7. Kondili E, Alexopoulou C, Xirouchaki N, Vaporidi K, Georgopoulos D. Estimation of inspiratory muscle pressure in critically ill patients. Intensive Care Med. 2010;36(4):648–55.

8. Younes M, Webster K, Kun J, Roberts D, Masiowski B. A Method for Measuring Passive Elastance during  Proportional Assist Ventilation. Am J Respir Crit Care Med. 2001;164(1):50–60.

9. Georgopoulos D, Prinianakis G, Kondili E. Bedside waveforms interpretation as a tool to identify patient-ventilator asynchronies. Intensive Care Med. 2006;32:34–47.

10. Vaporidi K, Akoumianaki E, Telias I, Goligher EC, Brochard L, Georgopoulos D. Respiratory Drive in Critically Ill Patients. Pathophysiology and Clinical Implications. Am J Respir Crit Care Med. 2020;201(1):20–32.

11. Euler C. Brainstem mechanisms for generation and control of breathing pattern. In: Handbook of Physiology. The Respiratory System. American Physiological Society, Bethesda, Maryland. 1986. P. 1–68.

12. Clark FJ, von Euler C. On the regulation of depth and rate of breathing. J Physiol. 1972;222(2):267–95.
