# Supplementary material for: Driving pressure of respiratory system and lung stress in mechanically ventilated patients with active breathing
Source: Crit Care. 2024 Jan 12;28:19. doi: 10.1186/s13054-024-04797-3 (PMC10785492; doi:10.1186/s13054-024-04797-3)
Supplement: Supplementary file 3 — Additional file 3. Individual data of Transpulmonary driving pressure over time. [file 13054_2024_4797_MOESM3_ESM.docx]

**Additional file 3**

**Driving pressure of respiratory system and lung stress**

**in mechanically ventilated patients with active breathing**

Figures S1-S31: Individual consecutive 7-breaths moving average measurements of transpulmonary driving pressure (ΔP_Lung_) during the recording period. Notice that only in patient no. 4 ΔP_Lung_ was ≥12 cmH_2_O during the whole recording period.
